# EphB1 controls long-range cortical axon guidance through a cell non-autonomous role in GABAergic cells

**DOI:** 10.1242/dev.201439

**Published:** 2024-02-28

**Authors:** Ahlem Assali, George Chenaux, Jennifer Y. Cho, Stefano Berto, Nathan A. Ehrlich, Christopher W. Cowan

**Affiliations:** ^1^ Department of Neuroscience, Medical University of South Carolina, Charleston, SC 29425, USA; ^2^ Department of Psychiatry, University of Texas Southwestern Medical School, Dallas, TX 75390, USA

**Keywords:** Axon guidance, EphB1, GABAergic long-range projections, Cerebral vasculature, Mouse

## Abstract

EphB1 is required for proper guidance of cortical axon projections during brain development, but how EphB1 regulates this process remains unclear. We show here that *EphB1* conditional knockout (cKO) in GABAergic cells (*Vgat*-Cre), but not in cortical excitatory neurons (*Emx1*-Cre), reproduced the cortical axon guidance defects observed in global *EphB1* KO mice. Interestingly, in *EphB1* cKO^Vgat^ mice, the misguided axon bundles contained co-mingled striatal GABAergic and somatosensory cortical glutamatergic axons. In wild-type mice, somatosensory axons also co-fasciculated with striatal axons, notably in the globus pallidus, suggesting that a subset of glutamatergic cortical axons normally follows long-range GABAergic axons to reach their targets. Surprisingly, the ectopic axons in *EphB1* KO mice were juxtaposed to major blood vessels. However, conditional loss of *EphB1* in endothelial cells (*Tie2*-Cre) did not produce the axon guidance defects, suggesting that EphB1 in GABAergic neurons normally promotes avoidance of these ectopic axons from the developing brain vasculature. Together, our data reveal a new role for EphB1 in GABAergic neurons to influence proper cortical glutamatergic axon guidance during brain development.

## Teaser

**Summary:** EphB1 controls long-range cortical axon guidance through a cell non-autonomous role in GABAergic cells. EphB1 is also required to prevent cortical and striatal axons from extending along developing ventral telencephalic vasculature.

## INTRODUCTION

In the developing nervous system, binding of EphB tyrosine kinase receptors to their cell surface-localized ephrin ‘ligands’ triggers bidirectional, intracellular signaling events that regulate proper axon guidance, cell migration, synapse formation and synapse plasticity (Kania and Klein, 2016). Previous studies have revealed deep-layer cortical axon guidance defects in the ventral telencephalon (VTel) of *EphB1*^−/−^ mice (Robichaux et al., 2014, 2016; Lodato et al., 2014), and these guidance errors were exacerbated in the *EphB1*^−/−^*;EphB2*^−/−^ mice, suggesting partial compensation by EphB2 receptors (Robichaux et al., 2014). EphB1 shows peak expression in cortical layers V and VI at mouse embryonic day (E) 15.5 (Robichaux et al., 2014; Lodato et al., 2014) – a time when long-range cortical axons are navigating toward their subcortical target zones (Grant et al., 2012). However, at this developmental stage, EphB1 is also expressed in the developing epithalamus (Robichaux et al., 2014), GABAergic cell progenitor structures (i.e. ganglionic eminences and the preoptic area), GABAergic interneurons migrating toward the developing cortex (Rudolph et al., 2014) and GABAergic spiny projection neurons (SPNs) of the developing striatum. Developing deep-layer cortical axons also express ephrin-Bs and navigate through EphB1-expressing brain regions in the VTel. As such, it was unclear whether EphB1 regulates cortical axon guidance in a cortical cell autonomous manner or whether it regulates long-range cortical glutamatergic projections indirectly via a key role in other cell populations.

To investigate the cell populations in which EphB1 regulates proper cortical axon guidance, we generated a new floxed *EphB1* mouse to allow for Cre-dependent *EphB1* loss-of-function analysis. Our findings reveal that EphB1 is required within vesicular GABA transporter (Vgat; also known as Slc32a1)-positive cells, but surprisingly not in glutamatergic cortical neurons, to control long-range cortical axon guidance in the VTel. Moreover, in *EphB1* knockout (KO) mice, we observed numerous aberrant subcortical axon bundles comprising both GABAergic striatal axons and glutamatergic cortical axons, suggesting that a subset of long-range cortical axons normally fasciculate along a subpopulation of long-range GABAergic axons to reach their proper target(s). Surprisingly, the misguided axons preferentially grew along major blood vessels in the VTel, suggesting that axonal EphB1 functions to repel navigating axons away from the developing blood vessels. These effects were not produced by *EphB1* loss-of-function in D1 or D2 dopamine receptor-expressing SPNs, Tie2-expressing vascular endothelial cells, suggesting that EphB1 functions in a subpopulation of Vgat-positive neurons to indirectly produce cortical axon guidance defects in apposition to developing striatal vasculature.

## RESULTS

### Cortical axon guidance defects in *EphB1* KO mice

**Fig. 1.**
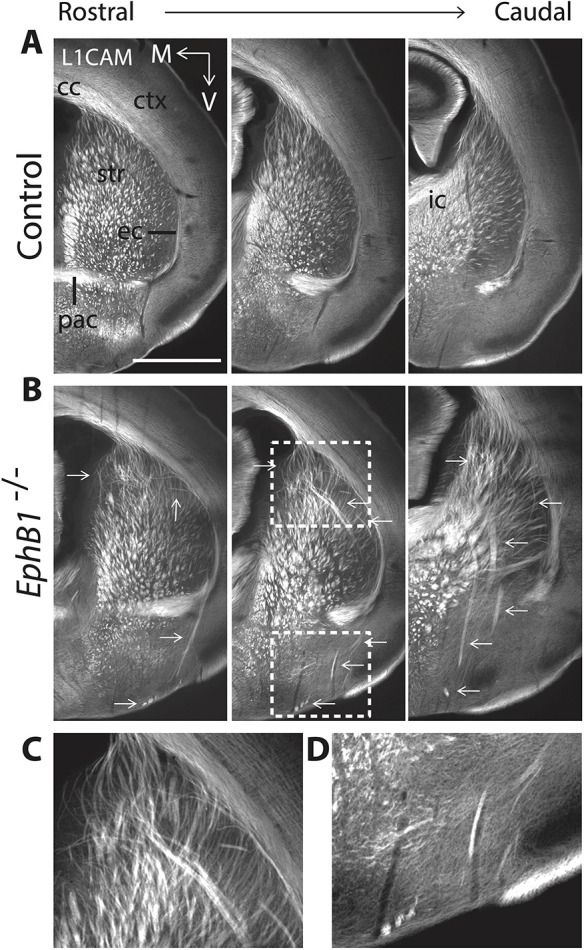
**Axon guidance defects in *EphB1*^−/−^ mice on a C57Bl6/J background.** (A,B) L1CAM staining on coronal sections at three different rostro-caudal levels at P0 in control mice (A) and in *EphB1*^−/−^ mice (B), with abnormal axon bundles in the dorsal striatum joining the external capsule (ec) and posterior branch of the anterior commissure (pac) and terminating in the ventral part of the brain, as well as abnormal axon bundles in the internal capsule (ic). Arrows indicate ectopic axon bundles. (C,D) Magnified images corresponding to the boxed areas in B. The images were taken using a microscope 10× objective. cc, corpus callosum; ctx, cortex; M, medial; str, striatum; V, ventral. *n*=5 *EphB1*^−/−^ and 8 control littermates. Scale bar: 1 mm (approximate).

As the background strain can influence phenotypes in mutant mice, we backcrossed the *EphB1*^−/−^ mice (on a CD-1 background; Williams et al., 2003) to C57BL/6 background strain (>8 generations). Similar to the *EphB1*^−/−^ on the CD-1 strain (Robichaux et al., 2014), the BL/6-backcrossed *EphB1*^−/−^ mice showed multiple axon guidance defects at postnatal day (P) 0 (Fig. 1), compared with wild-type mice (Fig. 1A), including disorganized axons and ectopic axon bundles within the dorsal striatum (Fig. 1B,C; see arrows), descending ectopic axon bundles within the internal capsule and ectopic axon projections descending from the external capsule and terminating near the brain floor (Fig. 1B,D; see arrows). Previous studies have also shown that axons from the somatosensory cortex aberrantly grow in the posterior branch of the anterior commissure in *EphB1*^−/−^ mice (Lodato et al., 2014).

### Loss of *EphB1* does not influence thalamic axon guidance

We previously reported subtle deficits in thalamic axon guidance in *EphB1*^−/−^*;EphB2*^−/−^ mice (Robichaux et al., 2014). As misguided VTel axons in *EphB1*^−/−^ mice originate largely from the cortex (Robichaux et al., 2014; Lodato et al., 2014) and ascending thalamocortical projections are thought to influence corticothalamic axon guidance (Molnar et al., 2012), we analyzed *Gbx2*-expressing thalamocortical projections in the *EphB1*^−/−^ mice. In the *EphB1*^−/−^ mice, the Gbx-GFP-labeled axon projections appeared to be normal (Fig. S1A,B), the L1CAM-positive misguided axons did not co-localize with GFP-positive thalamic fibers (Fig. S1C,C′) and the cortical barrel fields were similar to wild-type controls (Fig. S1D), suggesting that the cortical axon guidance errors in the VTel of *EphB1*^−/−^ mice are unlikely to be caused by aberrant thalamocortical axon navigation.

EphB1 is highly expressed in a dorsal region of the early developing thalamus (Robichaux et al., 2014). Careful analysis of EphB1 expression (using XGal staining) in the Gbx-GFP;EphB1-*lacZ* mice showed that EphB1 is highly expressed in the epithalamus (Fig. S2B), but largely undetectable in the developing thalamus (Fig. S2A). Indeed, the habenula-specific marker Brn3a (Pou4f1; Quina et al., 2009) revealed strong co-localization with EphB1 (X-gal) at E14.5, and EphB1 was highly expressed in the adult habenula (X-gal; Fig. S2B,C, respectively). Despite its strong expression in the habenula, we detected no axon guidance defects in the habenular commissure, the fasciculus retroflexus or the habenular axon tract in the interpeduncular nucleus in P0 *EphB1*^−/−^ pups (Fig. S2D). Together, these findings suggest that EphB1 is expressed highly in the developing epithalamus/habenula, but it is not required for normal habenular or thalamic axon guidance. The deficits in thalamic axon guidance in *EphB1*^−/−^*;EphB2*^−/−^ mice (Robichaux et al., 2014) might be due to the loss of *EphB2* or to a synergistic effect of the loss of both *EphB1* and *EphB2*.

### Generation of an *EphB1* conditional loss-of-function mutant mouse

To determine the cell population(s) in which *EphB1* functions to regulate proper cortical axon guidance, we generated a mutant mouse with loxP sites flanking *EphB1* exon 3 (*EphB1*^lox/lox^; Fig. S3) using traditional homologous recombination. To confirm that Cre-dependent loss of *EphB1* exon 3 reproduced the original *EphB1*^−/−^ phenotype, we generated germline transmission of the Δexon 3 allele by crossing *EphB1*^fl/fl^ mice with *Prm1*-Cre mice*.* After extensive backcrossing to C57BL/6, we confirmed the loss of *EphB1* expression in the brain of *EphB1*^ΔEx3/ΔEx3^ mice (Fig. S4A) and, importantly, we observed the same VTel axon guidance defects observed in the original *EphB1*^−/−^ mice (Fig. S4B).

### *EphB1* regulates cortical axon guidance via a role in GABAergic neurons

**Fig. 2.**
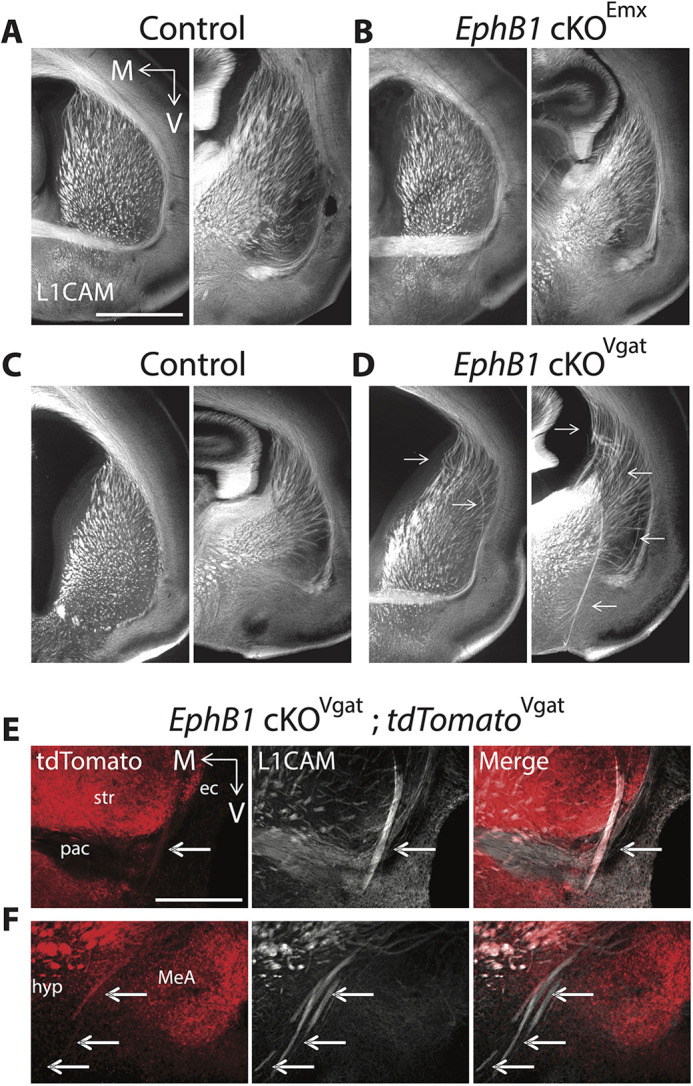
**Axon guidance defects after *EphB1* deletion from GABAergic cells.** (A-D) L1CAM staining on coronal sections at two different rostro-caudal levels at P0 in controls (A,C), in *EphB1* cKO^Emx^ (B) and in *EphB1* cKO^Vgat^ (D). *EphB1* deletion from GABAergic cells, but not glutamatergic cells, phenocopies the axon guidance defects (arrows) found in the global *EphB1*^−/−^ mice. (E,F) L1CAM and tdTomato co-staining on coronal sections at P0 in *EphB1* cKO^Vgat^;tdTomato^Vgat^ mice (Vgat-Cre×*EphB1*^lox/lox^×Ai14), showing that the misguided axon bundles include long-range GABAergic projections (arrows). The images were taken using a microscope 10× objective. ec, external capsule; hyp, hypothalamus; M, medial; MeA, medial amygdala nucleus; pac, posterior branch of the anterior commissure; str, striatum; V, ventral. *n*=4 *EphB1* cKO^Emx^ and 3 control littermates. *n*=5 *EphB1* cKO^Vgat^ and 5 control littermates. *n*=3 *EphB1* cKO^Vgat^;tdTomato^Vgat^. Scale bars: 1 mm (approximate).

As EphB1 is expressed in the deep layers of the developing cortex (Robichaux et al., 2014; Lodato et al., 2014), we crossed *EphB1*^lox/lox^ mice with Emx1*-*Cre mice to generate conditional *EphB1* KO (*EphB1* cKO^Emx1^) in most cortical and hippocampal glutamatergic pyramidal neurons starting at ∼E11.5 (Liang et al., 2012). Despite the loss of cortical EphB1 expression in the *EphB1* cKO^Emx1^ mice (Fig. S5A), we observed no cortical axon guidance defects (Fig. 2A,B). EphB1 is also highly expressed in multiple GABAergic neuron populations (Robichaux et al., 2014; Rudolph et al., 2014), including those within the developing VTel. To test EphB1 function in GABAergic neurons, we generated an *EphB1* cKO in virtually all GABAergic populations using *Vgat*-ires*-*Cre mice (Vong et al., 2011) (Fig. S5B-D). Interestingly, P0 *EphB1* cKO^Vgat^ pups completely phenocopied the cortical axon guidance defects found in the global *EphB1*^−/−^ pups (Fig. 2C,D). Together, these data revealed that EphB1 functions in GABAergic cells to influence cortical axon guidance.

### Cortical axons co-fasciculate with misguided striatal GABAergic axons in the absence of *EphB1*

**Fig. 3.**
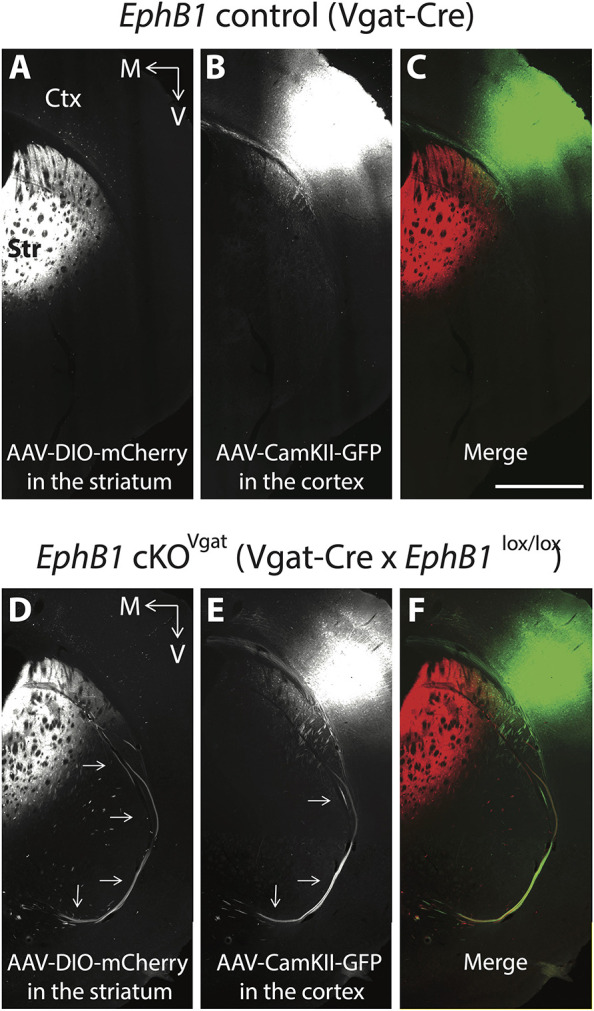
**Ectopic cortical glutamatergic projections intermingle with misguided striatal long-range GABAergic projections in *EphB1* cKO^Vgat^ mice.** (A-F) Ds-Red and GFP co-staining on coronal sections in adulthood in control mice (A-C) and *EphB1* cKO^Vgat^ mice (D-F) following Cre-dependent (DIO) mCherry AAV virus injections in the dorsal striatum (A,D) and CaMKII GFP AAV virus injections in the somatosensory cortex (B,E). (C,F) Merge. Arrows indicate the ectopic axons. The images were taken using a microscope 10× objective. Ctx, cortex; M, medial; Str, striatum; V, ventral. *N*=3 *EphB1* cKO^Vgat^ and 3 control littermates. Scale bar: 1 mm (approximate).

A subset of cortical GABAergic neurons [e.g. some parvalbumin (PV)- or somatostatin (Sst)-positive neurons] project long range to subcortical regions (Melzer and Monyer, 2020), suggesting a role of EphB1 in long-range projecting cortical GABAergic axon guidance. Using compound mutant mice *EphB1* cKO^Vgat^;tdTomato^Vgat^ (*EphB1*^lox/lox^×*Vgat*-Cre×Ai14), we indeed observed tdTomato-positive GABAergic long-range projecting axons located within the L1CAM-positive ectopic axon bundles at both P0 (Fig. 2E,F) and E15.5 (Fig. S6), suggesting that the GABAergic axon guidance deficits emerge as early as the misguided cortical axons. However, using a Cre-dependent virus approach in *EphB1* cKO*^Vgat^* mice, we did not detect Vgat-positive axons from the somatosensory cortex (i.e. the origin of many of the misprojected VTel axons in *EphB1*^−/−^; *EphB2*^−/−^ mice; Robichaux et al., 2014) within the ectopic axon bundles of *EphB1* cKO^Vgat^ mice (Fig. S7). These data suggest that the misguided GABAergic axons do not originate from long-range projecting cortical GABAergic neurons in the somatosensory cortex. GABAergic striatal SPNs in the striatum also project long range, and EphB1 is expressed in GABAergic cells of the developing and adult striatum (Rudolph et al., 2014; Fig. S5D). Using the same Cre-dependent virus approach in *EphB1* cKO*^Vgat^* mice, we clearly detected bundles of ectopic Vgat-positive cell axons originating from the dorsal striatum in the *EphB1* cKO^Vgat^ mice (Fig. 3D), compared with none seen in the control mice (Fig. 3A). To verify that misguided VTel axons in *EphB1* cKO*^Vgat^* mice also originate from cortical glutamatergic neurons, we used a virus labeling approach to label CaMKIIα-positive glutamatergic neurons in the somatosensory cortex. Indeed, in *EphB1* cKO*^Vgat^* mice, we observed misguided, CamKIIα-positive cortical glutamatergic axons in ectopic VTel axon bundles (Fig. 3E), compared with none seen in control mice (Fig. 3B). Importantly, in *EphB1* cKO*^Vgat^* mice, we detected co-fasciculation of ectopic Vgat-positive dorsal striatum axons and somatosensory cortex glutamatergic cell axons within the VTel ectopic axon bundles and in the posterior branch of the anterior commissure (Fig. 3F), compared with none seen in control mice (Fig. 3C). Together, these data revealed that EphB1 controls proper long-range glutamatergic cortical axon guidance through a cell non-autonomous role in GABAergic cells. Interestingly, in control mice, we also observed cortical glutamatergic axons co-mingled with striatal GABAergic axons along the rostro-caudal axis – specifically in the tracts projecting to the globus pallidus (Fig. S8A-D) and to the substantia nigra (Fig. S8E-H), two major targets of SPNs. These data strongly suggest that cortical glutamatergic axons traveling through the striatum fasciculate with striatal GABAergic axons to reach their proper targets.

### Loss of EphB1 in GABAergic D1 or D2 dopamine receptor-expressing populations does not phenocopy the axon guidance deficits observed in *EphB1* KO mice

**Fig. 4.**
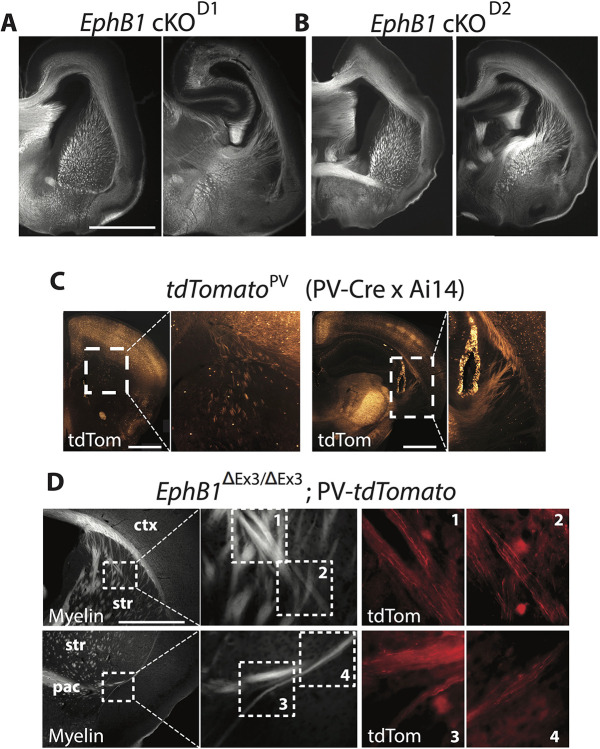
**No axon guidance defects after *EphB1* deletion from D1- or D2-SPNs, but misguided axons in EphB1-lacking mice include long-range projections from parvalbumin-positive neurons.** (A,B) L1CAM staining on coronal sections at two different rostro-caudal levels at P0 in *EphB1* cKO^D1^ (A) and in *EphB1* cKO^D2^ (B). (C) Ds-Red staining on coronal sections at two different rostro-caudal levels in adult tdTomato^PV^ mice (PV-Cre×Ai14), showing long-range projections from parvalbumin-positive neurons, including in the dorsal striatum. (D) Myelin and Ds-Red co-staining on different coronal sections in adult *EphB1*^ΔEx3/ΔEx3^;PV-tdTomato mice, showing that the misguided axon bundles include long-range projections from parvalbumin-positive neurons. The images were taken using a microscope 10× objective. Ctx, cortex; pac, posterior branch of the anterior commissure; Str, striatum; tdTom, tdTomato. *N*=3 *EphB1* cKO^D1^ and 3 control littermates. *N*=3 *EphB1* cKO^D2^ and 3 control littermates. *N*=3 *EphB1*^ΔEx3/ΔEx3^;PV-tdTomato. Scale bars: 1 mm (approximate).

The vast majority of striatal GABAergic cells are SPNs that express D1 or D2 dopamine receptors (Anderson et al., 2020). To test whether EphB1 might play a crucial role in D1- or D2-SPNs to produce the axon guidance defects, we first analyzed the effect of *EphB1*^−/−^ on D1- and D2-SPN projections in the *Drd1*-tdTomato or *Drd2*-GFP reporter mice. However, we failed to detect tdTomato-positive (D1) or GFP-positive (D2) axons within the VTel axon bundles in *EphB1*^−/−^ mice (Fig. S9A,B). Moreover, despite effective recombination in the *Drd1*-Cre and *Drd2*-Cre mice at E14.5 (Fig. S10A,B), conditional *EphB1* KO in either of these transgenic lines (i.e. *EphB1* cKO^D1^ and *EphB1* cKO^D2^) failed to produce the axon guidance deficits (Fig. 4A,B), suggesting that the VTel axon guidance phenotype in *EphB1*^−/−^ mice is not caused by a key role in developing D1- or D2-SPNs.

A subpopulation of Sst and PV GABAergic neurons are known to project long range from the cortex (Melzer and Monyer, 2020; Fig. 4C). PV neurons also project long range from the globus pallidus and substantia nigra (Saunders et al., 2016; Rodriguez and Gonzalez-Hernandez, 1999). In *EphB1^Δ^*^Ex3/ΔEx3^;PV-tdTomato mice, we detected long-range PV-positive axons within the misguided axon bundles (Fig. 4D). However, due to the absence of a PV-specific Cre line that recombines embryonically, we were not able to test the role of EphB1 specifically in PV neurons.

### Loss of EphB1 produces aberrant axon tracts along blood vessels in the ventral telencephalon

**Fig. 5.**
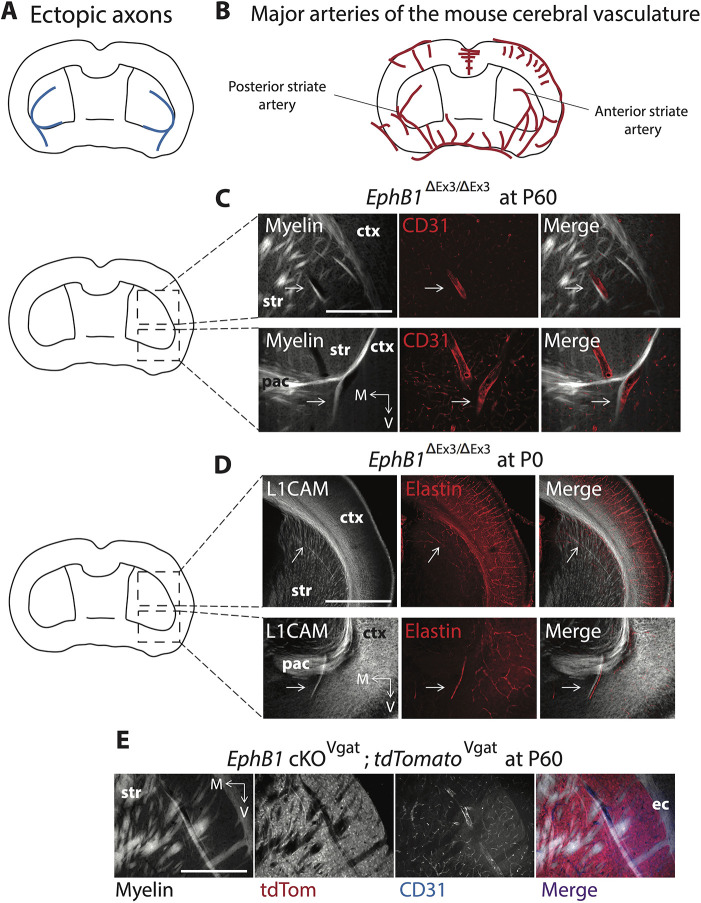
**Misguided axons juxtapose with major blood vessels in EphB1-lacking mice.** (A) Scheme representing the ectopic axons after EphB1 deletion. (B) Scheme representing major arteries of the mouse cerebral vasculature adapted from Dorr et al. (2007). (C) CD31 (endothelial cells marker) and myelin co-staining on different coronal sections in adult *EphB1*^ΔEx3/ΔEx3^ mice. (D) Elastin (arteries marker) and L1CAM co-staining on different coronal sections in P0 *EphB1*^ΔEx3/ΔEx3^ pups. Arrows (C,D) show misguided axons following blood vessels. (E) CD31, tdTomato and myelin co-staining on adult *EphB1* cKO^Vgat^;tdTomato^Vgat^ mice (Vgat-Cre×*EphB1*^lox/lox^×Ai14). ctx, cortex; ec, external capsule; M, medial; pac, posterior branch of the anterior commissure; str, striatum; V, ventral. The images were taken using a microscope 10× objective. *n*=3 *EphB1*^ΔEx3/ΔEx3^ at P60 (CD31). *n*=3 *EphB1*^ΔEx3/ΔEx3^ at P0 (elastin). *n*=3 *EphB1* cKO^Vgat^;tdTomato^Vgat^ at P60. Scale bars: 1 mm (approximate).

In the *EphB1*^ΔEx3/ΔEx3^ mice, we noticed that the VTel ectoptic axon fascicles were typically located in a similar anatomical location and pattern as VTel vasculature (Fig. 5A,B) (Dorr et al., 2007; Xiong et al., 2017; Kirst et al., 2020). Interestingly, the ectopic axon bundles in *EphB1*^ΔEx3/ΔEx3^ mice were observed in close apposition to large CD31 (Pecam1)-positive blood vessels (adults; Fig. 5C) and elastin-positive arteries (P0; Fig. 5D). In addition, long-range GABAergic projections were observed in the ectopic axon bundles located beside the striatal blood vessels (Fig. 5E).

**Fig. 6.**
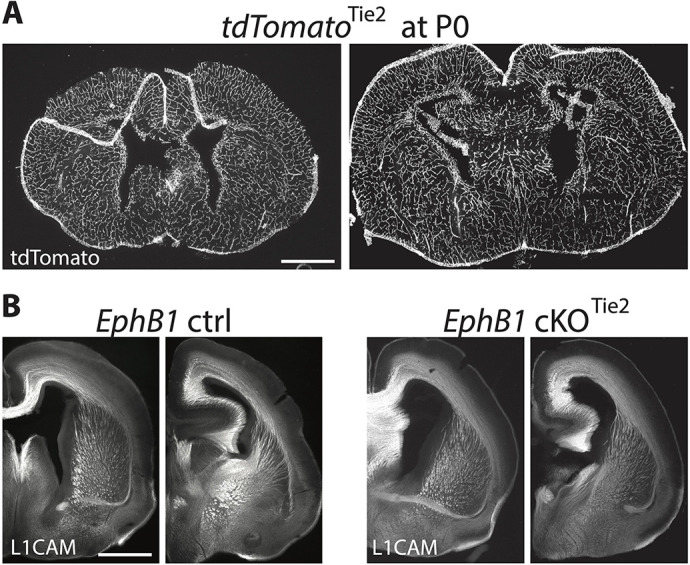
**No axon guidance defects after *EphB1* deletion from endothelial cells.** (A) Ds-Red staining on coronal sections at two different rostro-caudal levels in P0 tdTomato^Tie2^ pups (Tie2-Cre×Ai14). (B) L1CAM staining on coronal sections at two different rostro-caudal levels in P0 *EphB1* cKO^Tie2^ pups. The images were taken using a microscope 10× objective. *n*=3 *EphB1* cKO^Tie2^ and 3 control littermates. Scale bars: 1 mm (approximate).

Vascular endothelial cells express many axon guidance molecules, including members of the Eph/ephrin family (Fig. S11; Walchli et al., 2015; Adams et al., 1999; La Manno et al., 2021), that are required for proper vasculature development. Moreover, a subpopulation of vascular endothelial cells also express Vgat (Baruah and Vasudevan, 2019; Li et al., 2018), and EphB1 is expressed in angioblasts (i.e. endothelial cells precursor cells) at E14.5 (Fig. S11). To test whether EphB1 is required in Vgat-positive endothelial cells (instead of Vgat-positive GABAergic neurons) to possibly prevent ephrin-B-expressing GABAergic and glutamatergic axons (Fig. S11) from growing along developing blood vessels, we generated vascular endothelial cell-specific *EphB1* cKO^Tie2^ mice using *Tie2*-Cre mice (Kisanuki et al., 2001), where Cre expression in endothelial cells begins by ∼E13 (Li et al., 2018). However, although the *Tie2*-Cre mice showed specific brain vasculature recombination (Fig. 6A), the *EphB1* cKO^Tie2^ mice displayed no VTel axon guidance phenotypes (Fig. 6B). Taken together, these data suggest that EphB1 does not cause the cortical axon guidance deficits via a role in vascular endothelial cells, but rather EphB1 in GABAergic axons promotes avoidance of striatal, and indirectly cortical, axons from the developing brain vasculature (Fig. S12 and Table S1).

## DISCUSSION

To identify the cell populations in which EphB1 is required to regulate cortical long-range axon guidance, we generated and validated a new floxed *EphB1* mouse. This novel tool allowed us to show that, despite its expression in long-range glutamatergic cortical neurons, EphB1 functions in GABAergic cell populations, but surprisingly not in D1- or D2-receptor-expressing striatal SPNs, to influence correct cortical glutamatergic axon guidance in the developing striatum. We also detected striatal GABAergic axons co-fasciculated with cortical glutamatergic ectopic axon bundles, indicating that EphB1 is required for striatal cell long-range axon guidance, and suggesting that cortical glutamatergic axons are misrouted as an indirect consequence. The Eph/ephrin system is known to be involved in the proper migration of GABAergic neurons generated in the ganglionic eminences and preoptic area (Rudolph et al., 2014; Zimmer et al., 2008, 2011; Steinecke et al., 2014; Rudolph et al., 2010; Talebian et al., 2017); however, little was known about the role of the Eph/ephrin system in GABAergic axon guidance. Moreover, the absence of EphB1 in GABAergic cells causes the misguided VTel axons to navigate along the developing striatal vasculature, possibly owing to a failure of VTel axons to repel from ephrin-expressing vascular endothelial cells.

Based on the corticothalamic handshake hypothesis (Molnar et al., 2012), and our previous findings in *EphB1*^−/−^*;EphB2*^−/−^ mice showing deficits in both cortical and thalamic axon guidance (Robichaux et al., 2014), we speculated that the cortical axon guidance errors were an indirect effect of a role for EphB1 and EphB2 in ascending thalamocortical axons in the developing VTel. In the current study, we focused on the VTel axon guidance phenotypes in the single *EphB1*^−/−^ mouse, which were less severe than those seen in the *EphB1*^−/−^*;EphB2*^−/−^ mice (Robichaux et al., 2014). Using the Gbx-EGFP reporter mouse crossed to *EphB1*^−/−^ mice, we observed VTel axon guidance errors, but normal thalamocortical axon projections and no comingling of L1CAM-positive ectopic cortical axons with the GFP-positive thalamic axons. Careful examination revealed that EphB1 is highly expressed in the epithalamus (future habenula), but not detectably in the developing thalamus. However, no habenular axon guidance defects were observed in the *EphB1*^−/−^ mice. These findings suggest that the VTel axon guidance defects in *EphB1*^−/−^ mice are not likely to be caused by defects in thalamocortical axon guidance. However, EphB2 is expressed in the thalamus (Robichaux et al., 2014), so future studies in compound mutant mice will be required to investigate how EphB1 and EphB2 cooperate to influence proper thalamocortical axon guidance.

EphB1 mediates many of its biological functions through bidirectional signaling induced by contact-mediated binding to ephrins. EphrinB2 (Efnb2) is strongly expressed in the developing cortex and striatum (Robichaux et al., 2014), and we have previously shown that conditional deletion of *Efnb2* in *Nes*-Cre mice, where recombination includes cortical glutamatergic neurons, did not phenocopy the axon guidance deficits found in *EphB1*^−/−^ mice (Robichaux et al., 2014). Conditional deletion of *Efnb2* using the *FoxG1*-Cre line produced thalamo-cortical axon guidance deficits similar to those observed in *EphB1*^−/−^*;EphB2*^−/−^ mice, but did not phenocopy the cortical axon guidance deficits found in *EphB1*^−/−^ mice and in *EphB1*^−/−^*;EphB2*^−/−^ mice. EphrinB3, also expressed in the cortex and hippocampus, has been shown to control corticospinal tract axon guidance (Paixao et al., 2013), but future studies will be needed to assess its possible role in the *EphB1* cKO^Vgat^ phenotype.

Using viral-mediated axon tracing tools, we detected GABAergic axons intermingled with the misguided cortical axon bundles in the VTel and located along large blood vessels. As EphB1 is not required within excitatory cortical neurons for the axon guidance phenotypes but is necessary in Vgat, our findings suggest that EphB1 functions in a subset of GABAergic neurons to control proper long-range GABAergic axon guidance and avoidance of the developing VTel vasculature. Moreover, the cortical glutamatergic axon guidance defect is likely caused by co-fasciculation of the descending cortical glutamatergic neuron axons along the misrouted GABAergic fibers. As such, this suggested that a subset of long-range cortical axons might normally fasciculate along a subpopulation of long-range GABAergic axons to reach their proper target. Indeed, we found that, in control mice, somatosensory cortical glutamatergic axons co-fasciculated with striatal GABAergic axons along the rostro-caudal axis, specifically in the axon tracts projecting to the globus pallidus and substantia nigra. A recent study also showed that corticofugal axons fasciculate with striatal axons (Ehrman et al., 2022). In addition, a subpopulation of axons from the somatosensory and motor cortex projects to the globus pallidus (Karube et al., 2019), which is the predominant target of striatal SPNs. Moreover, the *EphB1*^−/−^ mice display a reduction in corticospinal tract axons (Lodato et al., 2014), suggesting that the misguided axons are largely corticofugal axons. In control mice, there is a subset of corticofugal axons that project to both the spinal cord and to striatal neurons via axon collaterals (Grillner and Robertson, 2016). Together, these observations strongly suggest that, in our model, a subset of cortical axons are misguided by following misrouted EphB1-null GABAergic striatal axons.

Although D1- and D2-SPNs are the predominant, long-range projecting GABAergic cell type in the striatum, conditional loss of *EphB1* in D1- or D2-dopamine-receptor-expressing cells failed to produce axon guidance defects, and no D1- or D2-positive axons were clearly observed in the ectopic bundles. We confirmed *Drd1*-Cre and *Drd2*-Cre recombination efficiency using a reporter mouse line (i.e. Ai14), so we cannot completely rule out the possibility that these Cre lines are less efficient at recombining the floxed EphB1 allele, but that explanation of our negative findings seems unlikely. It is also possible that a very small immature population of *Drd1*/*Drd2*-negative SPNs (Anderson et al., 2020) might produce the misrouted striatal axons labeled by the viral approach. Unpublished single-nuclei RNA-seq data in the mature striatum from our laboratory revealed two small subpopulations of *Grm8*-expressing SPNs that express very low or undetectable levels of D1 or D2 dopamine receptor mRNA (B. Hughes, S.B. and C.W.C., unpublished), so it is possible that these emerging ‘unconventional’ SPNs require EphB1 to control their axon guidance and give rise to the *EphB1* loss-of-function phenotypes. Sst-positive interneurons in the striatum can project over long distances within the striatum (Straub et al., 2016), but *EphB1* cKO^Sst^ failed to produce the *EphB1*^−/−^ phenotypes (A.A. and C.W.C., unpublished). Future studies validating EphB1 recombination in Sst-Cre mice will be necessary to confirm this negative result. In normally developing striatum, the PV-expressing GABAergic interneurons project at short-ranges (Straub et al., 2016), but it remains possible that loss of EphB1 causes striatal PV-positive cells to misproject. In addition, a subpopulation of PV-positive neurons (∼17%) in the globus pallidus projects to the striatum (Saunders et al., 2016), and loss of EphB1 in this population could misroute their axons within the striatum. Future studies examining EphB1 loss-of-function in various GABAergic subpopulations will be crucial for identifying the key GABAergic cell population(s) in which *EphB1* influences our observed axon guidance deficits in the *EphB1* KO^Vgat^ mice.

Of note, we found that a subset of striatal GABAergic and cortical glutamatergic axons in *EphB1*^ΔEx3/ΔEx3^ and *EphB1* cKO^Vgat^ are in close apposition to large blood vessels in the VTel. However, conditional loss of *EphB1* in *Tie2*-expressing vascular endothelial cells did not produce the noted axon guidance deficits, suggesting a role of EphB1 in GABAergic neurons. The developing vasculature and navigating axons express many of the same guidance molecules, including ephrins and Ephs (Walchli et al., 2015; Adams and Eichmann, 2010), suggesting that both systems can influence each other. However, the interplay between axon navigation and the developing vasculature has only begun to be examined in the central nervous system (Carmeliet and Tessier-Lavigne, 2005; Mukouyama et al., 2002; Mondo et al., 2020; Andreone et al., 2015). Several studies showed that some peripheral nerves (e.g. autonomic sympathetic axons) navigate along the vasculature via attractive cues expressed on, or secreted by, blood vessels (Mukouyama, 2014). Conversely, certain sensory nerves can provide a template to guide arterial patterning (Adams and Eichmann, 2010). Here, we add to this literature by showing that blood vessels also appear to influence striatal and cortical axon guidance. Except in the external capsule, cortical and striatal axons are generally not found in close apposition to the major blood vessels in the VTel, suggesting that EphB1 likely mediates the repulsion of these VTel axons from developing blood vessels. Using a single-cell RNA-seq dataset from whole embryonic brains at E14.5, we found that ephrinBs are expressed in numerous brain regions and cell types, including neurons, but also angioblasts, endothelial cells, mural cells and perivascular fibroblast-like cells. The arteriole endothelium strongly expresses *Efnb2* (Adams et al., 1999; Wang et al., 1998; Gerety et al., 1999), which could promote EphB1-dependent repulsion of navigating axons in the VTel. Unfortunately, loss of *Efnb2* from endothelial cells leads to early embryonic lethality (by E11.5) due to angiogenic defects (Adams et al., 1999), precluding our ability to examine whether loss of vascular *Efnb2* produces the VTel axon guidance phenotypes seen in the *EphB1*^−/−^ mice. Moreover, we cannot exclude the possibility that in the *EphB1* mutant mice, the colocalization of axons along the VTel blood vessels might be unrelated to the ability of EphB1 to mediate repulsive axon guidance. Together, our findings here reveal that EphB1 controls long-range cortical axon guidance through a cell non-autonomous role in one or more GABAergic cell populations during early brain development, and that the misguided cortical and striatal axons fascicles are observed in close apposition to the developing VTel vasculature.

## MATERIALS AND METHODS

### Animals

*EphB1* knockout mice (*EphB1*^−/−^) on a C57BL/6 background were generated by crossing *EphB1*^−/−^ mice on a CD-1 background (donated by Dr Henkemeyer, Williams et al., 2003) to C57BL/6 background strain (>8 generations). *EphB1*-*lacZ* mice were donated by Dr Henkemeyer (Chenaux and Henkemeyer, 2011). *EphB1*^ΔEx3/ΔEx3^ mice (*EphB1* total loss of function) were generated by crossing floxed *EphB1* mice (*EphB1*^lox/lox^, described below) to Prm-Cre mice (The Jackson Laboratory, #003328) to produce germline recombination. The Prm-Cre allele was subsequently removed during repeated backcrossing to C57BL/6J wild-type mice. *EphB1* cKO were generated by crossing *EphB1*^lox/lox^ mice with cell type-selective Cre-expressing transgenic mice (Emx1-Cre; The Jackson Laboratory, #005628), Vgat-Cre (The Jackson Laboratory, #028862), Drd1a-Cre (The Jackson Laboratory, #37156-JAX), Drd2-Cre (GENSAT; MMRRC, #036716-UCD), Tie2-Cre (The Jackson Laboratory, #008863), and they were compared with their Cre-negative littermate controls. The Vgat-Cre, Tie2-Cre, Drd1-Cre and Drd2-Cre lines were crossed to the Ai14 reporter mouse line (The Jackson Laboratory, #007914). To visualize long-range GABAergic projections, the Vgat-Cre line was crossed to both *EphB1*^lox/lox^ and Ai14 (*EphB1* cKO^Vgat^; tdTomato^Vgat^) mice or to Ai14 (tdTomato^Vgat^). To visualize thalamic and striatal axons in *EphB1* mice, *Gbx*-CreER^T2^-IRES-EGFP (The Jackson Laboratory, #022135), Drd1-tdTomato (The Jackson Laboratory, #016204) and *Drd2*-GFP (Mouse Genome Informatics, MGI:3843608) reporter mice were crossed to *EphB1*^−/−^ mice or to *EphB1*^ΔEx3/ΔEx3^ mice. To visualize the projections from PV-positive neurons, the parvalbumin-Cre (PV-Cre, The Jackson Laboratory, #017320) was crossed to the Ai14 mice (tdTomato^PV^), and the PV*-*tdTomato reporter line (The Jackson Laboratory, #027395) was crossed to *EphB1*^ΔEx3/ΔEx3^ mice (*EphB1*^ΔEx3/ΔEx3^;PV-tdTomato). All procedures were conducted in accordance with the Medical University of South Carolina Institutional Animal Care and Use Committee (IACUC) and National Institutes of Health guidelines.

### Generation of floxed EphB1 mutant mice (EphB^lox/lox^)

A targeting vector was generated by using a pL452 based mini targeting vector (Liu et al., 2003), which was recombined into mouse 129 strain bacterial artificial chromosome clone BMQ422J21 (Adams et al., 2005). The mini targeting vector was cloned so that loxP site #1 was inserted 722 bp upstream of EphB1 exon 3 and loxP site #2 was 293 bp downstream of exon 3. In addition to this, an FRT site flanked insertion was cloned downstream of loxP site #1, which contained the following cassettes: an engrailed two slice acceptor, the internal ribosomal entry site (IRES) from encephalomyocarditis virus (EMCV) (Bochkov and Palmenberg, 2006), a bovine tau protein and enhanced green fluorescent protein (eGFP) expression sequence (Utton et al., 2002), an SV40 polyadenylation signal and a neomycin selection cassette driven by prokaryotic and eukaryotic constitutive promoters. A capture vector was used to retrieve 10,630 bp for the left homology arm and 10,146 bp for the right homology arm with flanking diphtheria toxin and thymidine-kinase-negative selection cassettes, respectively. Murine stem cells were targeted and screened by the UC Davis Mouse Biology Program. Mice were crossed with Flp recombinase germline expression mice to remove the FRT flanked knock-in cassettes to generate *EphB1*^lox/lox^ mice lacking the selection cassettes. Cells that expressed Cre recombinase deleted 1854 bp of the *EphB1* genomic locus, which includes all of exon 3, to generate EphB1^Δexon3^ mice. Exons 1 and 2 have the potential to generate a small, truncated protein. In the *EphB1*^Δexon3^ mice, exon 2 splicing to exon 4 is predicted to produce either nonsense mediated decay or to code for the following protein sequence: MALDCLLLFLLASAVAAMEETLMDTRTATAELGWTANPASG**PVLRGPSRPARKLKAAPTAPPTVAPLQRRLPSAPAGLAITELTLIHQRWRVLVSHRVLEMSSPS** (bolded letters are produced by a frame-shift and represent the predicted non-EphB1 amino acids before the premature stop codon). The primers used to genotype floxed *EphB1* mice were: forward primer 5′-GGGAGAAGAGAGAGCCTAC-3′; reverse primer 5′-CCAGAGGGCTTTGAGTTAAT-3′ (floxed band: 316 bp; wild-type band: 420 bp). See Fig. S3.

### Immunohistochemistry

Adult mice were anesthetized with ketamine/xylazine diluted in 0.9% saline (120 mg/kg and 16 mg/kg, respectively) and hypothermia anesthesia was used for pups by placing them in ice for 5-8 min. P0 pups and adult mice were perfused transcardially with 4% (w/v) paraformaldehyde (PFA) in phosphate buffered saline (PBS), the brains were post-fixed overnight in 4% PFA, cryoprotected in 30% sucrose and then sectioned at 40 µm (adults) and 70 µm (pups) using a sliding microtome (Leica). Sections were washed in PBS, incubated in blocking solution [5% (v/v) normal donkey serum, 1% (w/v) bovine serum albumin, 0.2% (v/v) glycine, 0.2% (w/v) lysine, 0.3% (v/v) Triton X-100 in PBS] for 1 h at room temperature (RT) with shaking, incubated with primary antibodies diluted in blocking solution overnight at 4°C under shaking and then washed three times for 10 min in PBS with gentle shaking at RT. Sections were then incubated with secondary antibodies diluted in blocking solution for 90 min at RT with shaking and protected from light, washed three times for 10 min in PBS and mounted in ProLong Gold Antifade Mountant (Invitrogen, #P36931). Antibodies used were: rat anti-L1CAM (1:1000, Millipore Sigma, #MAB5272); chicken anti-GFP (1:1000, Abcam, #13970); mouse anti-Brn3a (1:200, Thermo Fisher Scientific, #MAB1585); rabbit anti-DsRed for tdTomato staining (1:1000, Living Colors, #632496); rabbit anti-RFP for tdTomato staining (1:3000, Rockland, #600-401-379); goat anti-CD31 (1:800, Novus Biologicals, #AF3628); mouse anti-elastin (1:800, Sigma-Aldrich, #MAB2503); Cy3 donkey anti-rat (1:500, Thermo Fisher Scientific, #NC0236073); AlexaFluor 488 donkey anti-mouse (1:500, Thermo Fisher Scientific, #NC0192065); AlexaFluor 488 donkey anti-chicken (1:500, Thermo Fisher Scientific, #703-545-155).

### XGal staining

Adult mice were perfused transcardially with 4%(w/v) PFA, and the brains were post-fixed overnight in 4% (w/v) PFA. Female mice were bred and vaginal plugs were assessed with the day of plug detection considered as E0.5. After live-decapitation, embryo brains were drop-fixed in 4% PFA overnight. Adult and embryo brains were then cryoprotected in 30% sucrose. Embryo brains were plunged into M1-embedding matrix (Thermo Fisher Scientific, #1310), flash-frozen for 1 min in isopentane between −20°C and −30°C, and stored at −80°C. Adult brains were cut at 40 µm using a sliding microtome (Leica) and embryo brains at 20 µm using a cryostat (Leica). XGal staining was performed using the beta-galactosidase staining kit (Mirus, MIR 2600), following the manufacturer's instructions. Briefly, the sections were washed in PBS, incubated in the Cell Staining Working Solution containing the X-Gal Reagent in a dark, humidified chamber at 37°C overnight, washed once in PBS and mounted in ProLong Gold Antifade Mountant (Invitrogen, #P36931).

### Myelin stain

Sections were stained for myelin using the BrainStain Imaging kit (Thermo Fisher Scientific, #B34650; FluoroMyelin, 1:300), following the manufacturer's instructions.

### RT-PCR

RNA extraction was performed using the miRNeasy Mini kit (Qiagen, #1038703), following the manufacturer's instructions. Total RNA was reverse-transcribed using Superscript III (Invitrogen) with random hexamers, following the manufacturer's instructions. PCRs were performed using the complementary DNA to detect *EphB1* expression (for *EphB1* cKO^Emx^: forward primer 5′-TACAGAGATGCGCTT-3′, reverse primer 5′-ACAGCGTGGCCTGCA-3′; for *EphB1^ΔEx3/ΔEx3^* and for *EphB1* cKO^Vgat^: forward primer 5′-AGACATTGATGGACACAAGG-3′, reverse primer 5′-TCAAAGTCAGCTCGGTAATA-3′). GAPDH was used as a control (forward primer 5′-TGAAGGTCGGTGTCAACGGATTTGGC-3′; reverse primer 5′-CATGTAGGCCATGAGGTCCACCAC-3′).

### RNAscope^®^

After live-decapitation, the brains were plunged in M1-embedding matrix (Thermo Fisher Scientific, #1310), flash-frozen for 1 min in isopentane between −20°C and −30°C, stored at −80°C, and then cut at 16 µm thick slices using a cryostat (Leica). Sections were kept at −20°C during the cutting process and stored at −80°C. RNAscope^®^ was performed using the RNAscope^®^ kit (ACD Bio, #323110) and following the ACD protocol provided by the manufacturer. Sections were immersed in 4% (w/v) PFA for 15 min, then in 50% (v/v) ethanol for 5 min, then in 70% (v/v) ethanol for 5 min, and then twice in 100% ethanol for 5 min. Sections were covered by RNAscope^®^ hydrogen peroxide for 10 min in a humidified chamber and washed three times in PBS, and the protease incubation step was omitted. Sections were placed in a humidified chamber in the HybEZ™ Oven for all the following steps at 40°C and were washed twice for 2 min in wash buffer after each of the following incubation steps. Sections were incubated with the probes (ACD EphB1 custom probe designed in EphB1 exon3 #541171-C2; Vgat probe #319191; tdTomato probe #317041-C3; C1 probes were used without dilution; C2 and C3 probes were diluted 50× in C1 probe or in diluent) and placed at 40°C for 2 h. AMP1, AMP2 and AMP3 were successively applied on the sections for 30 min each at 40°C. Sections were then covered by the appropriate horseradish peroxidase isoenzyme C and placed at 40°C for 15 min, covered by the appropriate fluorophore [PerkinElmer, #NEL744E001KT (Cyanine3), #NEL741E001KT (Fluorescein), #NEL745E001KT (Cyanine5); 1:2000 in TSA Buffer provided in the RNAScope^®^ kit], placed at 40°C for 30 min, covered by the HRP blocker and incubated at 40°C for 15 min. Finally, DAPI was applied for 1 min and sections were mounted in ProLong Gold Mountant (Invitrogen, #P36931).

### Stereotaxic injections

Unilateral stereotaxic injections of AAV5-CaMKIIα-EGFP (Addgene plasmid #50469; virus titer ≥3×10^12^ vg/ml) and AAV5-hSyn-DIO-hM4D(Gi)-mCherry (Addgene plasmid #44362; virus titer ≥7×10^12^ vg/ml) viruses were performed on adult anesthetized (isoflurane) control (Vgat-Cre) and *EphB1* cKO^Vgat^ (*EphB1*^lox/lox^×Vgat-Cre) mice, into the dorsal striatum (DV: −2.8, ML: +1.6, AP: 0; 150 nl) and into two locations of the somatosensory cortex [(1) DV: −1.9, ML: +3.2, AP: −0.4; (2) DV: −1.4, ML: +2.7, AP: −1.7; 200 nl in each location], using a Nanoinjector (Thermo Fisher Scientific, #13-681-455; 50 nl/30 s). Placement was confirmed by immunohistochemistry (GFP and DsRed antibodies; see above).

### Bioinformatics analysis of a single cell RNA-seq dataset from whole mouse embryonic brain

Single-cell gene expression data were downloaded from http://mousebrain.org/ (La Manno et al., 2021). Briefly, loom files were converted into Seurat objects with UMI data and metadata. Downstream analysis was performed in R with Seurat (v4.1.0) (Hao et al., 2021) and customized R scripts. Data were preprocessed, normalized and filtered. The Seurat function DotPlot was used to visualize the gene expression and relative abundance of the genes of interest across the different cell subclasses. Cell information was stored in the metadata.

## Supplementary Material



10.1242/develop.201439_sup1 Supplementary information

## References

[DEV201439C1] Adams, R. H. and Eichmann, A. (2010). Axon guidance molecules in vascular patterning. *Cold Spring Harb. Perspect. Biol.* 2, a001875. 10.1101/cshperspect.a001875 20452960 PMC2857165

[DEV201439C2] Adams, R. H., Wilkinson, G. A., Weiss, C., Diella, F., Gale, N. W., Deutsch, U., Risau, W. and Klein, R. (1999). Roles of ephrinB ligands and EphB receptors in cardiovascular development: demarcation of arterial/venous domains, vascular morphogenesis, and sprouting angiogenesis. *Genes Dev.* 13, 295-306. 10.1101/gad.13.3.295 9990854 PMC316426

[DEV201439C3] Adams, D. J., Quail, M. A., Cox, T., van der Weyden, L., Gorick, B. D., Su, Q., Chan, W., Davies, R., Bonfield, J. K., Law, F. et al. (2005). A genome-wide, end-sequenced 129Sv BAC library resource for targeting vector construction. *Genomics.* 86, 753-758. 10.1016/j.ygeno.2005.08.003 16257172

[DEV201439C4] Anderson, A. G., Kulkarni, A., Harper, M. and Konopka, G. (2020). Single-cell analysis of Foxp1-driven mechanisms essential for striatal development. *Cell Rep.* 30, 3051-3066.e7. 10.1016/j.celrep.2020.02.030 32130906 PMC7137930

[DEV201439C5] Andreone, B. J., Lacoste, B. and Gu, C. (2015). Neuronal and vascular interactions. *Annu. Rev. Neurosci.* 38, 25-46. 10.1146/annurev-neuro-071714-033835 25782970 PMC5729758

[DEV201439C6] Baruah, J. and Vasudevan, A. (2019). The vessels shaping mental health or illness. *Open Neurol. J.* 13, 1-9. 10.2174/1874205X01913010001 30984305 PMC6460472

[DEV201439C7] Bochkov, Y. A. and Palmenberg, A. C. (2006). Translational efficiency of EMCV IRES in bicistronic vectors is dependent upon IRES sequence and gene location. *BioTechniques* 41, 283-284, 286, 288 passim. 10.2144/000112243 16989088

[DEV201439C8] Carmeliet, P. and Tessier-Lavigne, M. (2005). Common mechanisms of nerve and blood vessel wiring. *Nature* 436, 193-200. 10.1038/nature03875 16015319

[DEV201439C9] Chenaux, G. and Henkemeyer, M. (2011). Forward signaling by EphB1/EphB2 interacting with ephrin-B ligands at the optic chiasm is required to form the ipsilateral projection. *Eur. J. Neurosci.* 34, 1620-1633. 10.1111/j.1460-9568.2011.07845.x 22103419 PMC3228319

[DEV201439C10] Dorr, A., Sled, J. G. and Kabani, N. (2007). Three-dimensional cerebral vasculature of the CBA mouse brain: a magnetic resonance imaging and micro computed tomography study. *Neuroimage* 35, 1409-1423. 10.1016/j.neuroimage.2006.12.040 17369055

[DEV201439C11] Ehrman, J. M., Merchan-Sala, P., Ehrman, L. A., Chen, B., Lim, H.-W., Waclaw, R. R. and Campbell, K. (2022). Formation of the mouse internal capsule and cerebral peduncle: a pioneering role for striatonigral axons as revealed in Isl1 conditional mutants. *J. Neurosci.* 42, 3344-3364. 10.1523/JNEUROSCI.2291-21.2022 35273083 PMC9034787

[DEV201439C12] Gerety, S. S., Wang, H. U., Chen, Z.-F. and Anderson, D. J. (1999). Symmetrical mutant phenotypes of the receptor EphB4 and its specific transmembrane ligand ephrin-B2 in cardiovascular development. *Mol. Cell* 4, 403-414. 10.1016/S1097-2765(00)80342-1 10518221

[DEV201439C13] Grant, E., Hoerder-Suabedissen, A. and Molnár, Z. (2012). Development of the corticothalamic projections. *Front. Neurosci.* 6, 53. 10.3389/fnins.2012.00053 22586359 PMC3343305

[DEV201439C14] Grillner, S. and Robertson, B. (2016). The basal ganglia over 500 million years. *Curr. Biol.* 26, R1088-R1100. 10.1016/j.cub.2016.06.041 27780050

[DEV201439C15] Hao, Y., Hao, S., Andersen-Nissen, E., Mauck, W. M., Zheng, S., Butler, A., Lee, M. J., Wilk, A. J., Darby, C., Zager, M. et al. (2021). Integrated analysis of multimodal single-cell data. *Cell* 184, 3573-3587.e29. 10.1016/j.cell.2021.04.048 34062119 PMC8238499

[DEV201439C16] Kania, A. and Klein, R. (2016). Mechanisms of ephrin-Eph signalling in development, physiology and disease. *Nat. Rev. Mol. Cell Biol.* 17, 240-256. 10.1038/nrm.2015.16 26790531

[DEV201439C17] Karube, F., Takahashi, S., Kobayashi, K. and Fujiyama, F. (2019). Motor cortex can directly drive the globus pallidus neurons in a projection neuron type-dependent manner in the rat. *eLife* 8, e49511. 10.7554/eLife.49511 31711567 PMC6863630

[DEV201439C18] Kirst, C., Skriabine, S., Vieites-Prado, A., Topilko, T., Bertin, P., Gerschenfeld, G., Verny, F., Topilko, P., Michalski, N., Tessier-Lavigne, M. et al. (2020). Mapping the fine-scale organization and plasticity of the brain vasculature. *Cell* 180, 780-795.e25. 10.1016/j.cell.2020.01.028 32059781

[DEV201439C19] Kisanuki, Y. Y., Hammer, R. E., Miyazaki, J.-, Williams, S. C., Richardson, J. A. and Yanagisawa, M. (2001). Tie2-Cre transgenic mice: a new model for endothelial cell-lineage analysis in vivo. *Dev. Biol.* 230, 230-242. 10.1006/dbio.2000.0106 11161575

[DEV201439C20] La Manno, G., Siletti, K., Furlan, A., Gyllborg, D., Vinsland, E., Mossi Albiach, A., Mattsson Langseth, C., Khven, I., Lederer, A. R., Dratva, L. M. et al. (2021). Molecular architecture of the developing mouse brain. *Nature* 596, 92-96. 10.1038/s41586-021-03775-x 34321664

[DEV201439C21] Li, S., Kumar T, P., Joshee, S., Kirschstein, T., Subburaju, S., Khalili, J. S., Kloepper, J., Du, C., Elkhal, A., Szabó, G. et al. (2018). Endothelial cell-derived GABA signaling modulates neuronal migration and postnatal behavior. *Cell Res.* 28, 221-248. 10.1038/cr.2017.135 29086765 PMC5799810

[DEV201439C22] Liang, H., Hippenmeyer, S. and Ghashghaei, H. T. (2012). A Nestin-cre transgenic mouse is insufficient for recombination in early embryonic neural progenitors. *Biol. Open.* 1, 1200-1203. 10.1242/bio.20122287 23259054 PMC3522881

[DEV201439C23] Liu, P., Jenkins, N. A. and Copeland, N. G. (2003). A highly efficient recombineering-based method for generating conditional knockout mutations. *Genome Res.* 13, 476-484. 10.1101/gr.749203 12618378 PMC430283

[DEV201439C24] Lodato, S., Molyneaux, B. J., Zuccaro, E., Goff, L. A., Chen, H.-H., Yuan, W., Meleski, A., Takahashi, E., Mahony, S., Rinn, J. L. et al. (2014). Gene co-regulation by Fezf2 selects neurotransmitter identity and connectivity of corticospinal neurons. *Nat. Neurosci.* 17, 1046-1054. 10.1038/nn.3757 24997765 PMC4188416

[DEV201439C25] Melzer, S. and Monyer, H. (2020). Diversity and function of corticopetal and corticofugal GABAergic projection neurons. *Nat. Rev. Neurosci.* 21, 499-515. 10.1038/s41583-020-0344-9 32747763

[DEV201439C26] Molnar, Z., Garel, S., López-Bendito, G., Maness, P. and Price, D. J. (2012). Mechanisms controlling the guidance of thalamocortical axons through the embryonic forebrain. *Eur. J. Neurosci.* 35, 1573-1585. 10.1111/j.1460-9568.2012.08119.x 22607003 PMC4370206

[DEV201439C27] Mondo, E., Becker, S. C., Kautzman, A. G., Schifferer, M., Baer, C. E., Chen, J., Huang, E. J., Simons, M. and Schafer, D. P. (2020). A developmental analysis of juxtavascular microglia dynamics and interactions with the vasculature. *J. Neurosci.* 40, 6503-6521. 10.1523/JNEUROSCI.3006-19.2020 32661024 PMC7486666

[DEV201439C28] Mukouyama, Y. S. (2014). Vessel-dependent recruitment of sympathetic axons: looking for innervation in all the right places. *J. Clin. Invest.* 124, 2855-2857. 10.1172/JCI76622 24937419 PMC4071382

[DEV201439C29] Mukouyama, Y.-S., Shin, D., Britsch, S., Taniguchi, M. and Anderson, D. J. (2002). Sensory nerves determine the pattern of arterial differentiation and blood vessel branching in the skin. *Cell* 109, 693-705. 10.1016/S0092-8674(02)00757-2 12086669

[DEV201439C30] Paixao, S., Balijepalli, A., Serradj, N., Niu, J., Luo, W., Martin, J. H. and Klein, R. (2013). EphrinB3/EphA4-mediated guidance of ascending and descending spinal tracts. *Neuron* 80, 1407-1420. 10.1016/j.neuron.2013.10.006 24360544 PMC4030410

[DEV201439C31] Quina, L. A., Wang, S., Ng, L. and Turner, E. E. (2009). Brn3a and Nurr1 mediate a gene regulatory pathway for habenula development. *J. Neurosci.* 29, 14309-14322. 10.1523/JNEUROSCI.2430-09.2009 19906978 PMC2802832

[DEV201439C32] Robichaux, M. A., Chenaux, G., Ho, H.-Y. H., Soskis, M. J., Dravis, C., Kwan, K. Y., Šestan, N., Greenberg, M. E., Henkemeyer, M. and Cowan, C. W. (2014). EphB receptor forward signaling regulates area-specific reciprocal thalamic and cortical axon pathfinding. *Proc. Natl. Acad. Sci. USA* 111, 2188-2193. 10.1073/pnas.1324215111 24453220 PMC3926086

[DEV201439C33] Robichaux, M. A., Chenaux, G., Ho, H. Y. H., Soskis, M. J., Greenberg, M. E., Henkemeyer, M. and Cowan, C. W. (2016). EphB1 and EphB2 intracellular domains regulate the formation of the corpus callosum and anterior commissure. *Dev. Neurobiol.* 76, 405-420. 10.1002/dneu.22323 26148571 PMC5473157

[DEV201439C34] Rodriguez, M. and Gonzalez-Hernandez, T. (1999). Electrophysiological and morphological evidence for a GABAergic nigrostriatal pathway. *J. Neurosci.* 19, 4682-4694. 10.1523/JNEUROSCI.19-11-04682.1999 10341266 PMC6782595

[DEV201439C35] Rudolph, J., Zimmer, G., Steinecke, A., Barchmann, S. and Bolz, J. (2010). Ephrins guide migrating cortical interneurons in the basal telencephalon. *Cell Adh. Migr.* 4, 400-408. 10.4161/cam.4.3.11640 20473036 PMC2958617

[DEV201439C36] Rudolph, J., Gerstmann, K., Zimmer, G., Steinecke, A., Döding, A. and Bolz, J. (2014). A dual role of EphB1/ephrin-B3 reverse signaling on migrating striatal and cortical neurons originating in the preoptic area: should I stay or go away? *Front. Cell Neurosci.* 8, 185. 10.3389/fncel.2014.00185 25100946 PMC4103172

[DEV201439C37] Saunders, A., Huang, K. W. and Sabatini, B. L. (2016). Globus Pallidus Externus neurons expressing parvalbumin interconnect the subthalamic nucleus and striatal interneurons. *PLoS ONE* 11, e0149798. 10.1371/journal.pone.0149798 26905595 PMC4764347

[DEV201439C38] Steinecke, A., Gampe, C., Zimmer, G., Rudolph, J. and Bolz, J. (2014). EphA/ephrin A reverse signaling promotes the migration of cortical interneurons from the medial ganglionic eminence. *Development* 141, 460-471. 10.1242/dev.101691 24381199

[DEV201439C39] Straub, C., Saulnier, J. L., Bègue, A., Feng, D. D., Huang, K. W. and Sabatini, B. L. (2016). Principles of synaptic organization of GABAergic interneurons in the striatum. *Neuron* 92, 84-92. 10.1016/j.neuron.2016.09.007 27710792 PMC5074692

[DEV201439C40] Talebian, A., Britton, R., Ammanuel, S., Bepari, A., Sprouse, F., Birnbaum, S. G., Szabó, G., Tamamaki, N., Gibson, J. and Henkemeyer, M. (2017). Autonomous and non-autonomous roles for ephrin-B in interneuron migration. *Dev. Biol.* 431, 179-193. 10.1016/j.ydbio.2017.09.024 28947178 PMC5658245

[DEV201439C41] Utton, M. A., Connell, J., Asuni, A. A., van Slegtenhorst, M., Hutton, M., de Silva, R., Lees, A. J., Miller, C. C. J. and Anderton, B. H. (2002). The slow axonal transport of the microtubule-associated protein tau and the transport rates of different isoforms and mutants in cultured neurons. *J. Neurosci.* 22, 6394-6400. 10.1523/JNEUROSCI.22-15-06394.2002 12151518 PMC6758152

[DEV201439C42] Vong, L., Ye, C., Yang, Z., Choi, B., Chua, S. and Lowell, B. B. (2011). Leptin action on GABAergic neurons prevents obesity and reduces inhibitory tone to POMC neurons. *Neuron* 71, 142-154. 10.1016/j.neuron.2011.05.028 21745644 PMC3134797

[DEV201439C43] Walchli, T., Wacker, A., Frei, K., Regli, L., Schwab, M. E., Hoerstrup, S. P., Gerhardt, H. and Engelhardt, B. (2015). Wiring the vascular network with neural cues: a CNS perspective. *Neuron* 87, 271-296. 10.1016/j.neuron.2015.06.038 26182414

[DEV201439C44] Wang, H. U., Chen, Z.-F. and Anderson, D. J. (1998). Molecular distinction and angiogenic interaction between embryonic arteries and veins revealed by ephrin-B2 and its receptor Eph-B4. *Cell* 93, 741-753. 10.1016/S0092-8674(00)81436-1 9630219

[DEV201439C45] Williams, S. E., Mann, F., Erskine, L., Sakurai, T., Wei, S., Rossi, D. J., Gale, N. W., Holt, C. E., Mason, C. A. and Henkemeyer, M. (2003). Ephrin-B2 and EphB1 mediate retinal axon divergence at the optic chiasm. *Neuron.* 39, 919-935. 10.1016/j.neuron.2003.08.017 12971893

[DEV201439C46] Xiong, B., Li, A., Lou, Y., Chen, S., Long, B., Peng, J., Yang, Z., Xu, T., Yang, X., Li, X. et al. (2017). Precise cerebral vascular atlas in stereotaxic coordinates of whole mouse brain. *Front. Neuroanat.* 11, 128. 10.3389/fnana.2017.00128 29311856 PMC5742197

[DEV201439C47] Zimmer, G., Garcez, P., Rudolph, J., Niehage, R., Weth, F., Lent, R. and Bolz, J. (2008). Ephrin-A5 acts as a repulsive cue for migrating cortical interneurons. *Eur. J. Neurosci.* 28, 62-73. 10.1111/j.1460-9568.2008.06320.x 18662335

[DEV201439C48] Zimmer, G., Rudolph, J., Landmann, J., Gerstmann, K., Steinecke, A., Gampe, C. and Bolz, J. (2011). Bidirectional ephrinB3/EphA4 signaling mediates the segregation of medial ganglionic eminence- and preoptic area-derived interneurons in the deep and superficial migratory stream. *J. Neurosci.* 31, 18364-18380. 10.1523/JNEUROSCI.4690-11.2011 22171039 PMC6623906

