# Supplementary material for: EphB1 controls long-range cortical axon guidance through a cell non-autonomous role in GABAergic cells
Source: Development. 2024 Feb 28;151(5):dev201439. doi: 10.1242/dev.201439 (PMC10946438; doi:10.1242/dev.201439)
Supplement: Supplementary information [file develop-151-201439-s1.pdf]

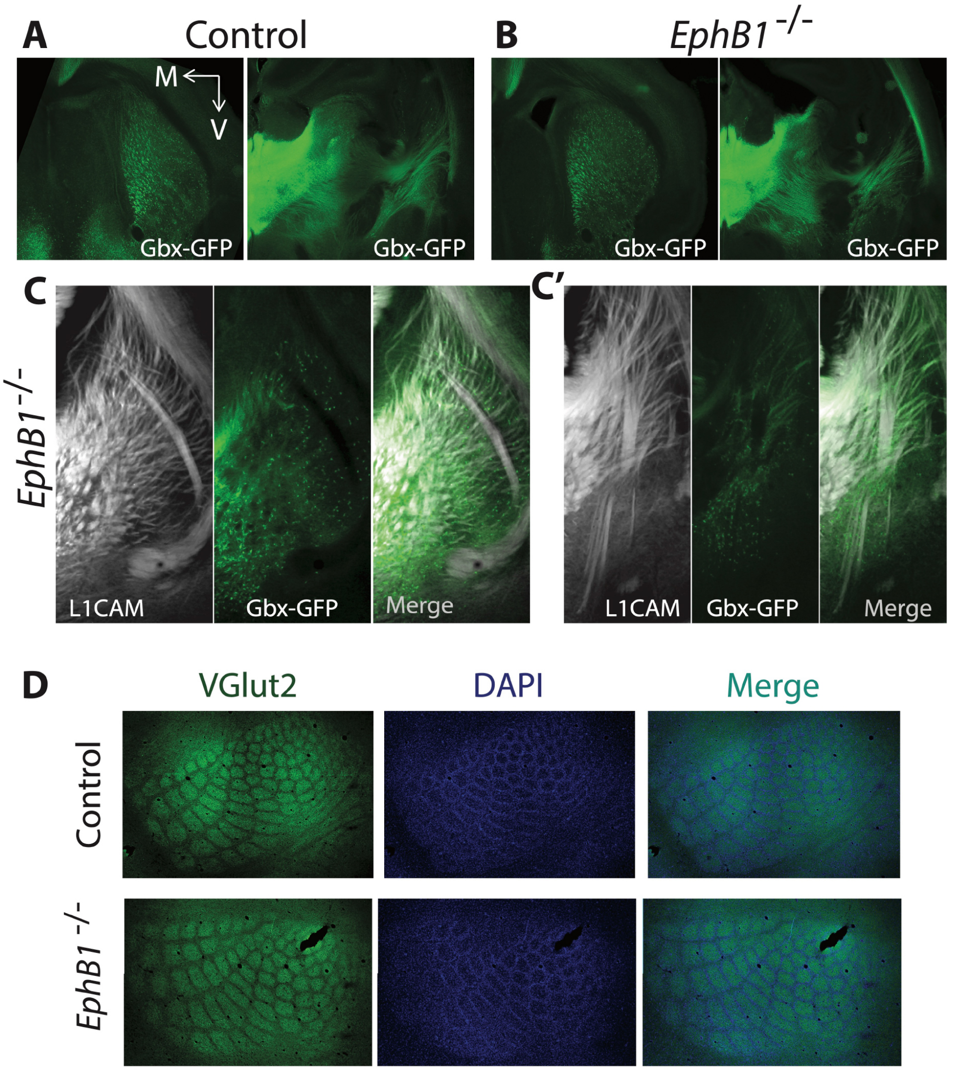

Fig. S1. No misguided thalamic projections in EphB1$^{-/-}$ mice. A, B. GFP staining of thalamic nuclei and projections on coronal sections at two different rostro-caudal levels at PO in Gbx-GFP control **(A)** and in Gbx-GFP; *EphB1$^{-/-}$* pups **(B). C, C'.** L1CAM and GFP co-staining on coronal sections at two different rostro-caudal levels in PO Gbx-GFP; *EphB$^{-/-}$* pups. n = 6 Gbx-GFP; *EphB1$^{-/-}$* and 7 control littermates.**D.** Vglut2 and DAPI co-staining on tangential sections of flattened barrel cortex in adult control and *EphB1$^{-/-}$* mice. n = 3 *EphB1$^{-/-}$* and 4 control littermates. The images were taken using a microscope 10X objective. V: ventral; M: medial.

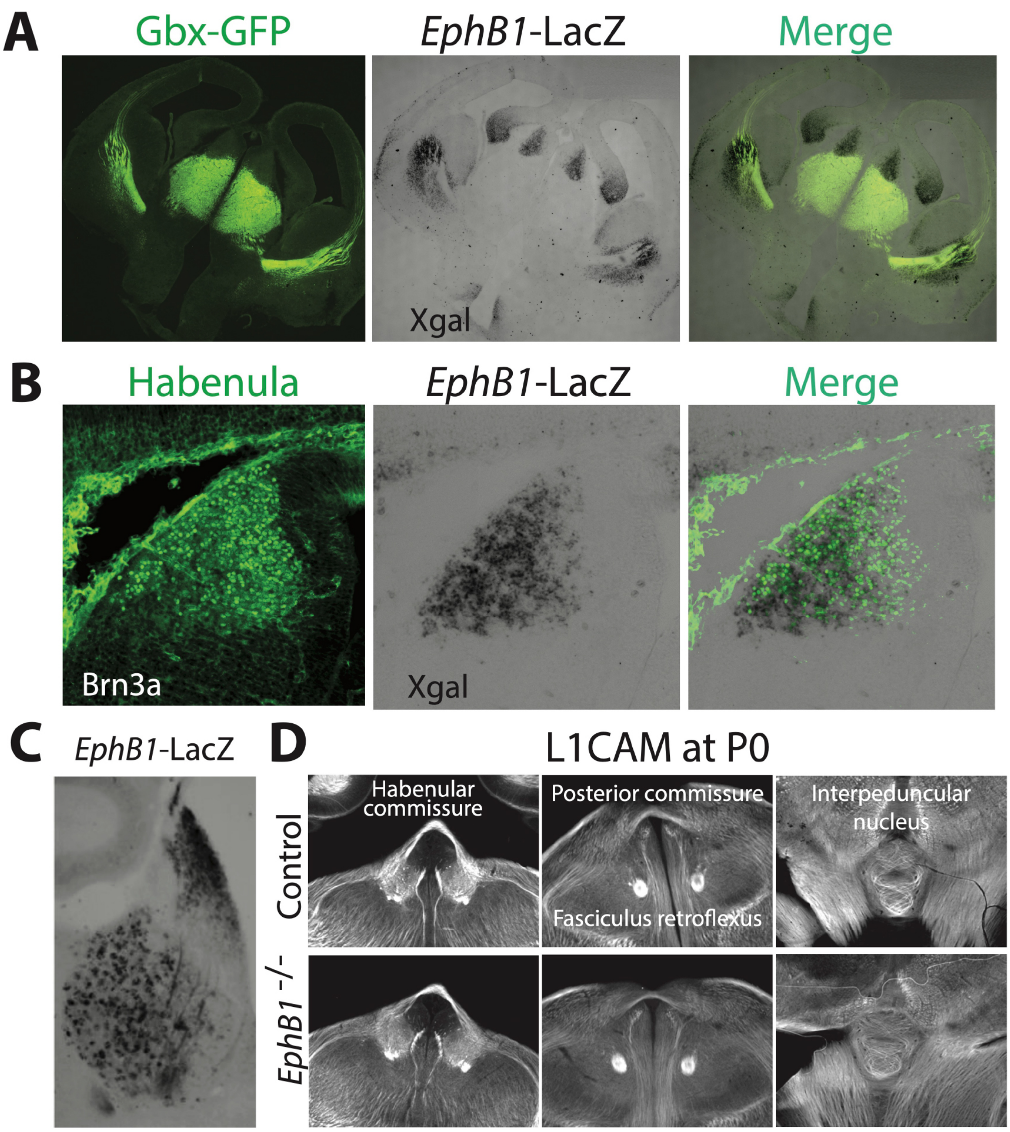

Fig. S2. EphB1 expression in the habenula. A. GFP and Xgal co-staining on coronal sections at E14.5 in Gbx-GFP;*EphB1*-LacZ embryos. **B.** Brn3a (marker of developing habenula) and Xgal co-staining on coronal sections of the habenula at E14.5 in *EphB1*-LacZ embryos. **C.** Xgal staining on coronal sections of the habenula in adult *EphB1*-LacZ mice. **D.** L1CAM staining on coronal sections of habenular axon tracts at PO in control (upper panel in **D)** and *EphB1*$^{-/-}$ mice (lower panel in **D).** The images were taken using a microscope 10X objective.

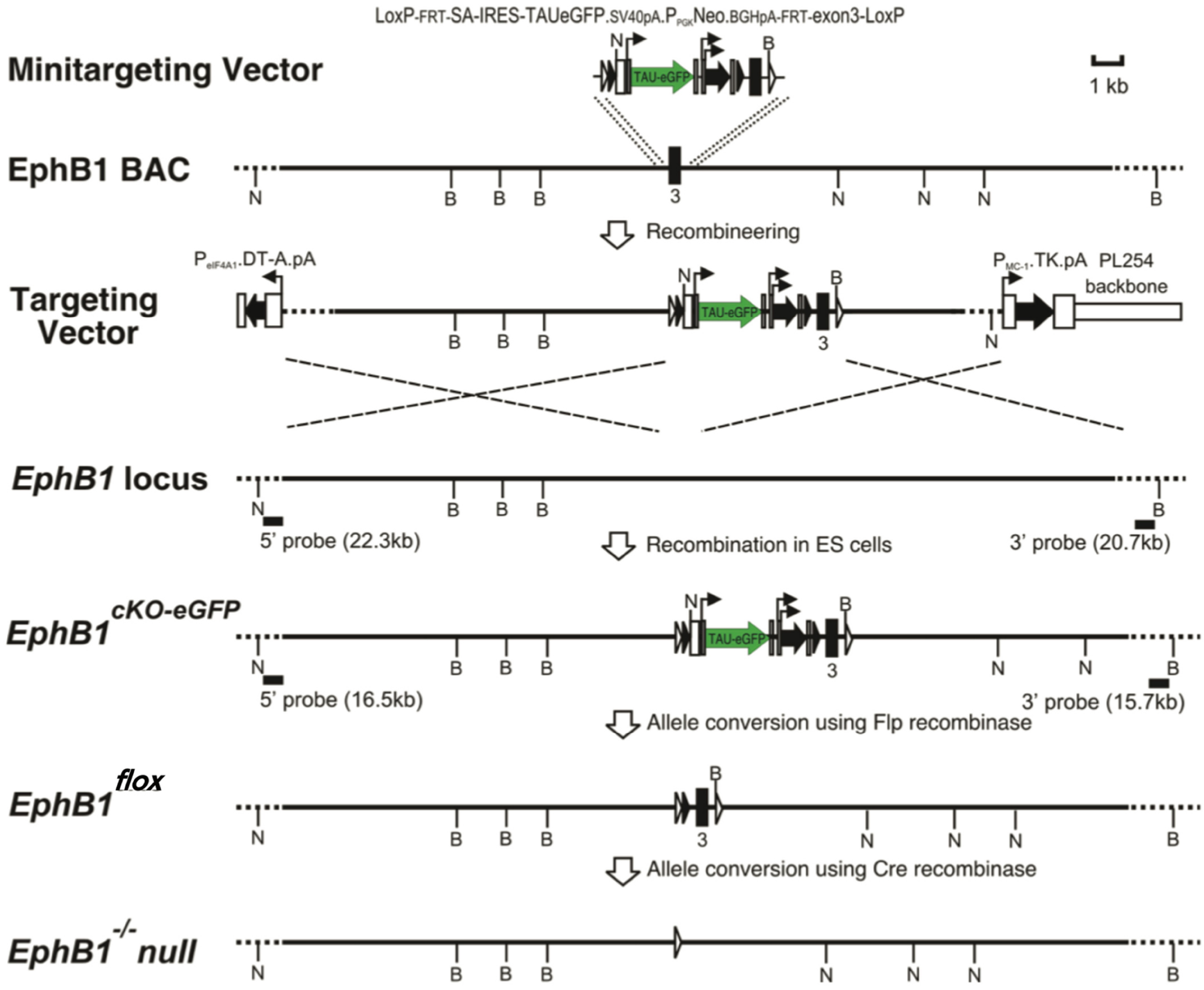

Fig. S3. Generation of floxed EphB1 mice. All details are in the materials and methods section.

Development • Supplementary information

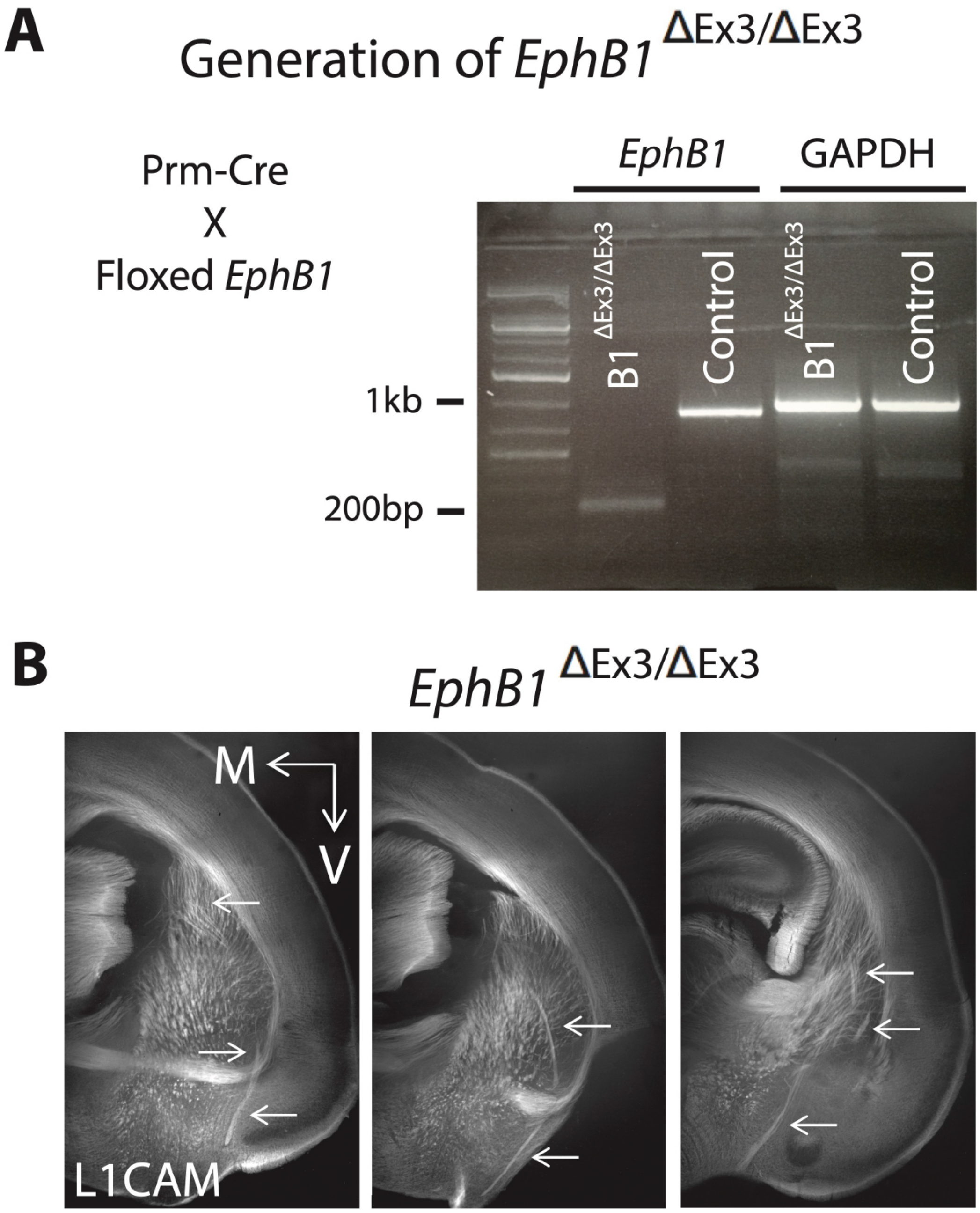

Fig. S4. Generation and validation of floxed EphB1 mice.

A. Generation of a novel global *EphB1* knockout mouse. *EphB1*$^{lox/lox}$ mice were crossed to Prm-Cre mice to generate germline transmission of the *EphB1* loss-of-function allele (*EphB1*$^{\Delta Ex3/\Delta Ex3}$). RT-PCR showing efficient recombination of *EphB1* exon 3 in *EphB1*$^{\Delta Ex3/\Delta Ex3}$ mice compared to control mice (excised exon 3 band: 153bp; control band: 880bp). GAPDH was used as a control. **B.** L1CAM staining on coronal sections at three different rostro-caudal levels at PO on *EphB1*$^{\Delta Ex3/\Delta Ex3}$ mice, showing the same axon guidance defects (arrows) as in global EphB1$^{-/-}$ mice. The images were taken using a microscope 10X objective.V: ventral; M: medial.

Development • Supplementary information

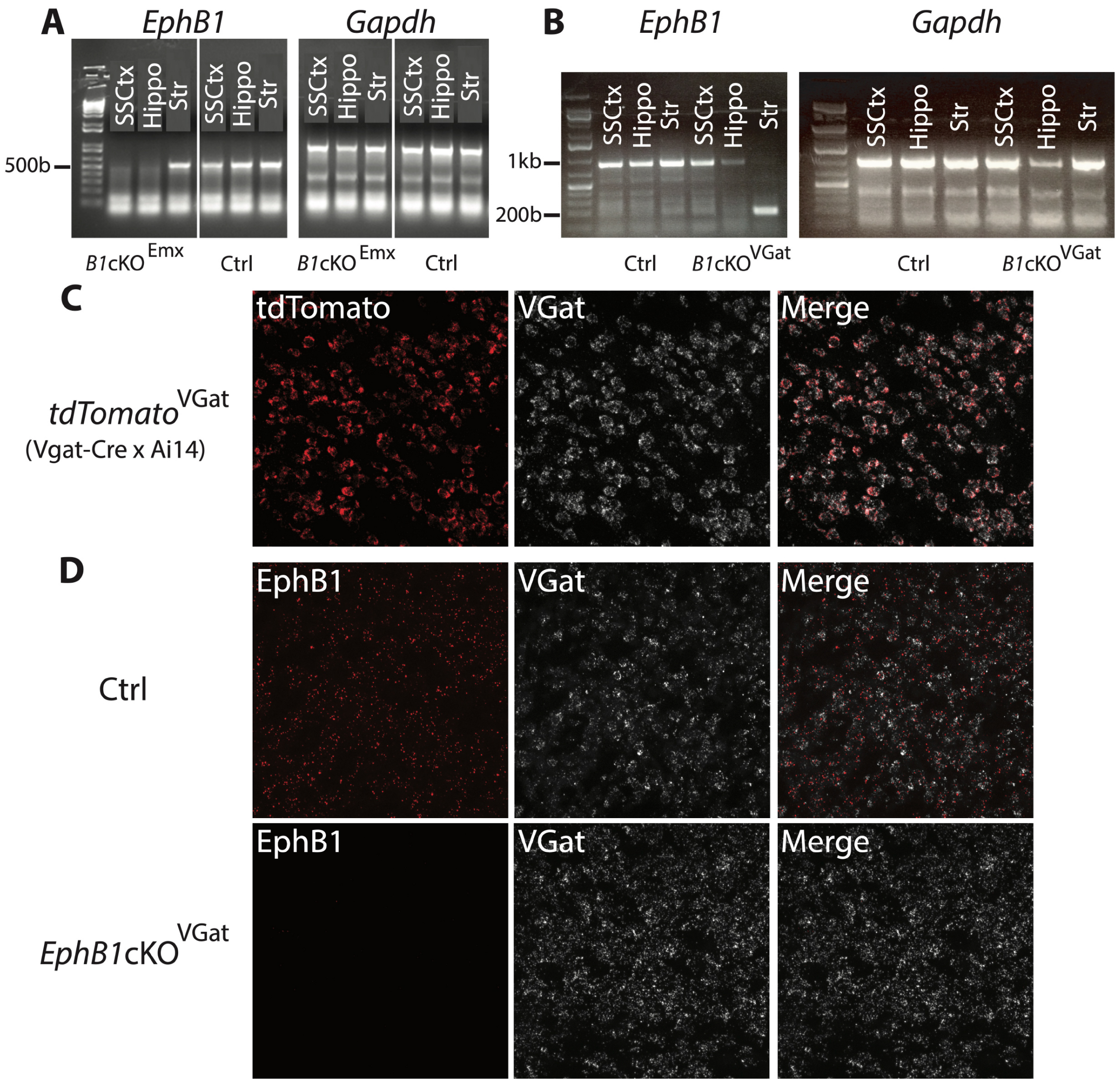

Fig. S5. EphB1 deletion validation in EphB1 cKO^{Emx} and in EphB1 cKo^{vgat} mice. **A, B.** RT-PCR showing specific recombination of *EphB1* exon 3 in *EphB1* cKQ^{Emx} mice (excised exon 3: no band;control band: 545bp) (**A**) and in *EphB1* cKo^{Vgat} mice (excised exon 3 band: 153bp; control band: 880bp) (**B**) compared to control mice.The two sets of primers for detection are described in the materials and methods section. *Gapdh* was used as a control. SSCtx:somatosensory cortex; Hippo: hippocampus; Str:striatum. **C.** tdTomato and Vgat co-staining using fluorescent in situ hybridization (RNAscope) on coronal sections of the dorsal striatum in *tdTomato*^{Vgat} mice, showing perfect colocalization between tdTomato and endogenous Vgat. **D.** EphB1 and Vgat co-staining using RNAscope on coronal sections of the dorsal striatum in *tdTomato*^{Vgat} control mice (upper panel in **D**) and in *tdTomato*^{Vgat}; *EphB1* cKO^{Vgat} mice (lower panel in ^{D}), showing loss of *EphB1* expression in Vgat-positive cells after *EphB1* deletion. The images were taken using a confocal 20X objective.

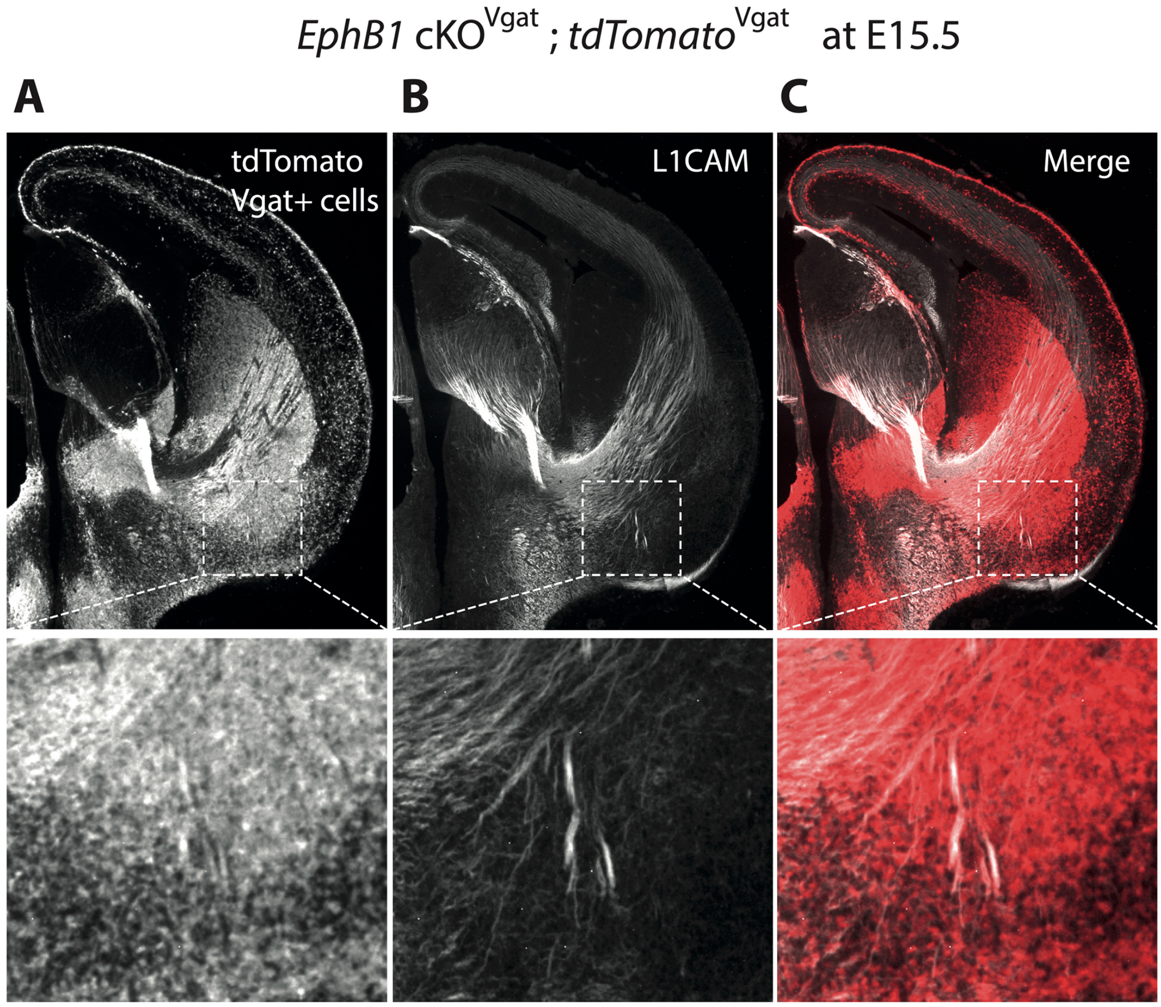

Fig. S6. Vgat positive misguided axons at E15.5. Ds-Red (**A**) and L1CAM (**B**) co-staining on coronal sections in *EphB1* cKo^{Vgat}; *tdTomato*^{Vgat} mice (Vgat-Cre x *EphB1*$^{lox/lox}$ x Ai14) at E15.5, showing Vgat positive misguided axons at E15.5 among L1CAM positive axons. The images in the lower panel are a zoom of the images in the white squares of the upper panel. n = 2 *EphB1* cKo^{Vgat}; *tdTomato*^{Vgat}. The images were taken using a microscope 10X objective.

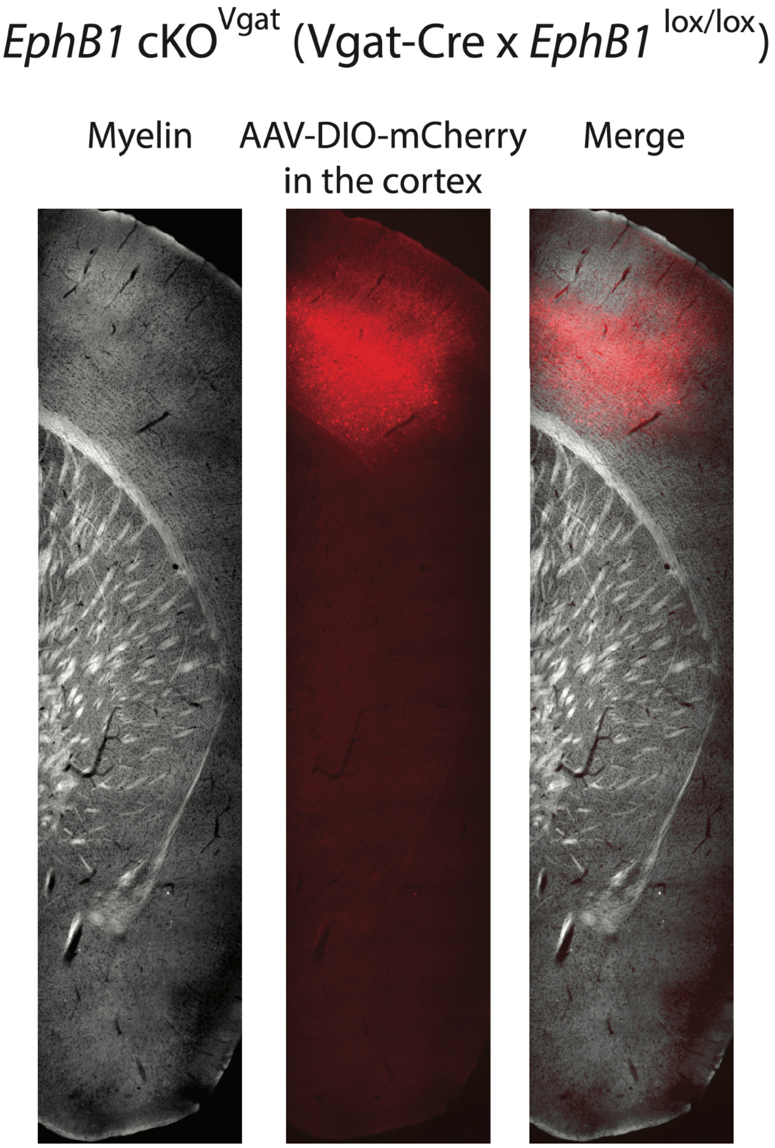

Fig. S7. No misguided cortical long-range GABAergic projections in EphB1 cKOVgat mice. Myelin and Ds-Red co-staining on coronal sections of adult *EphB1 cKo*^{Vgat} mice, following Cre-dependent (DIO) mCherry AAV virus injections in the somatosensory cortex. n = *EphB1* cKo^{Vgat}. The images were taken using a microscope 10X objective.

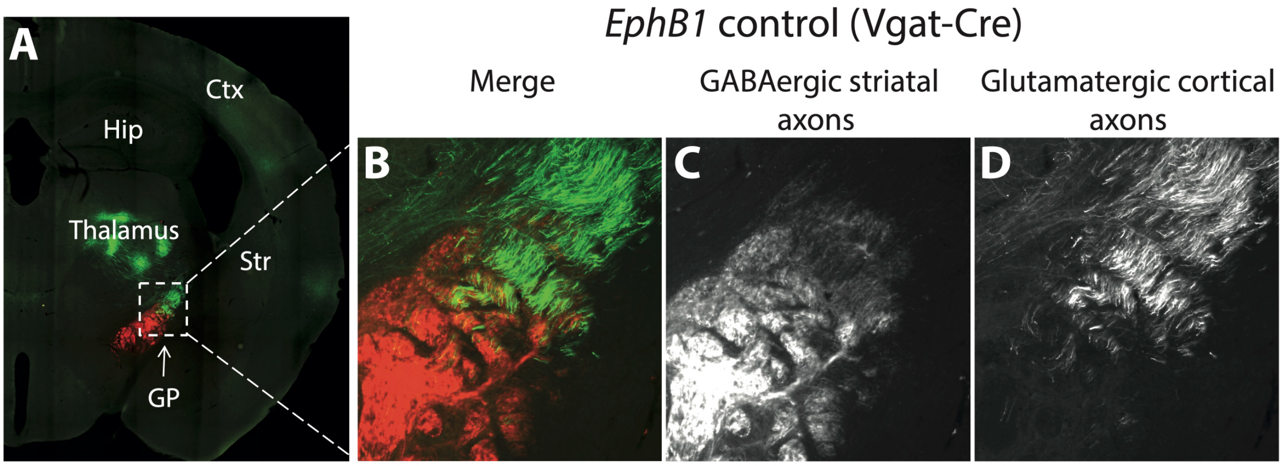

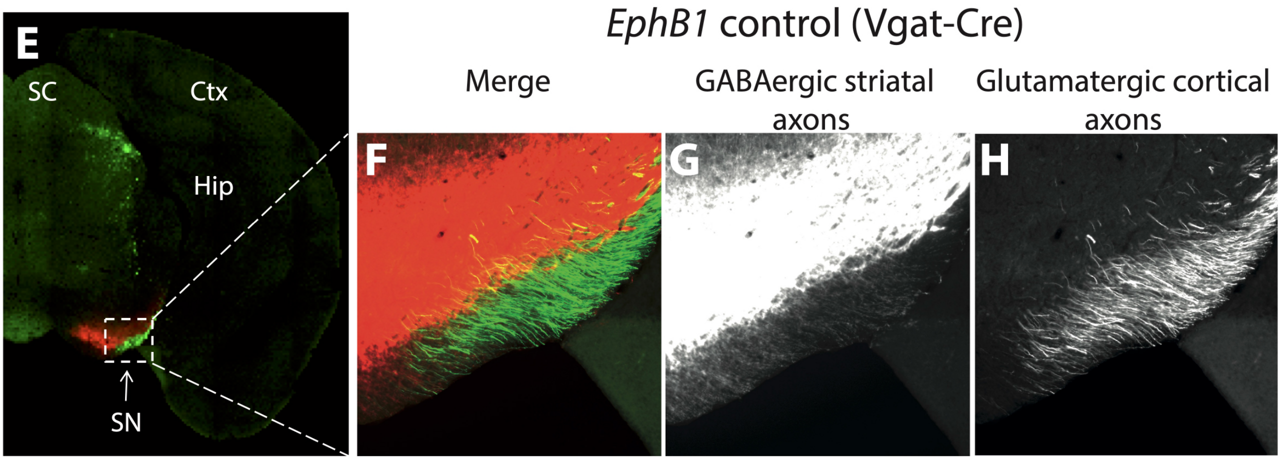

Fig. S8. Cofasciculation of striatal GABAergic and somato-sensory-cortical glutamatergic axons. Ds-Red **(C, G)** and GFP **(D, H)** co-staining on coronal sections in control mice (Vgat-Cre mice), at the level of the globus pallidus **(A-D)** and of the substantia nigra **(E-H),** following (re-dependent (DIO) mCherry AAV virus injections in the dorsal striatum and CaMKll GFP AAV virus injections in the somatosensory cortex. n = 3 Vgat-Cre. The images were taken using a microscope 10X objective. Ctx: cortex; Hip: hippocampus; Str: striatum; GP: globus pallidus; SC: superior colliculus; SN: substantia nigra.

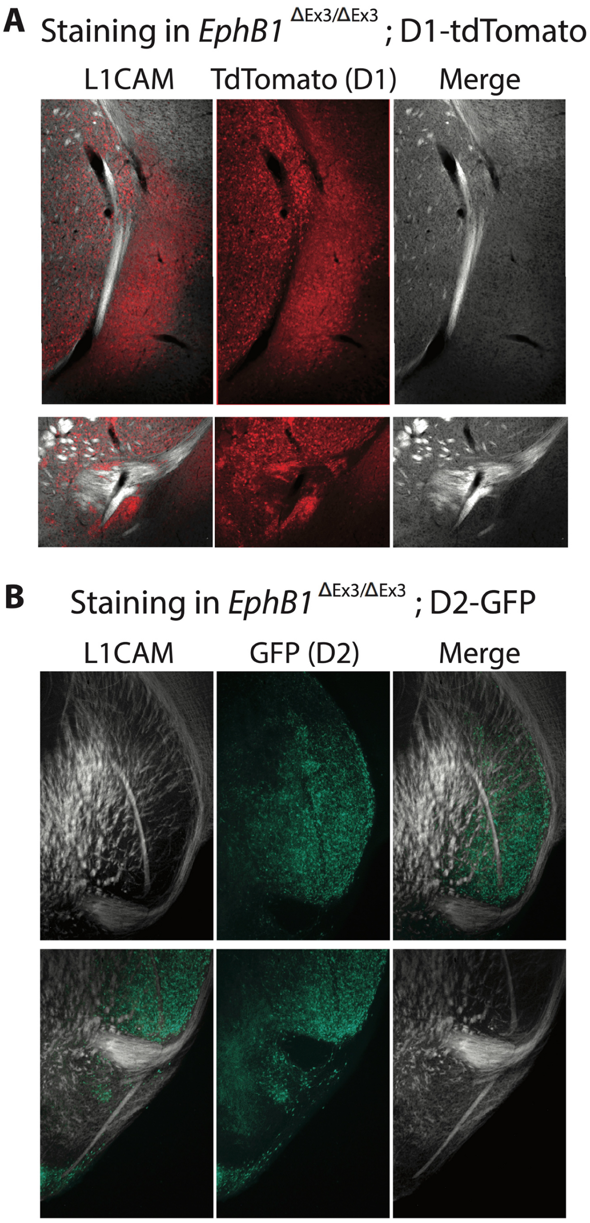

Fig. S9. No clear misguided axons from D1- and D2-SPNs in EphB1$^{\Delta Ex3/\Delta Ex3}$ mice. A. Myelin and Os-Red co-staining on coronal sections of adult D1-td-*Tomato;EphB1*$^{\Delta Ex3/\Delta Ex3}$ mice. **B.** L1CAM and GFP co-staining on coronal sections of *D2-GFP; EphB1*$^{\Delta Ex3/\Delta Ex3}$ pups at P0. The images were taken using a microscope 10X objective. V: ventral; M: medial.

Development • Supplementary information

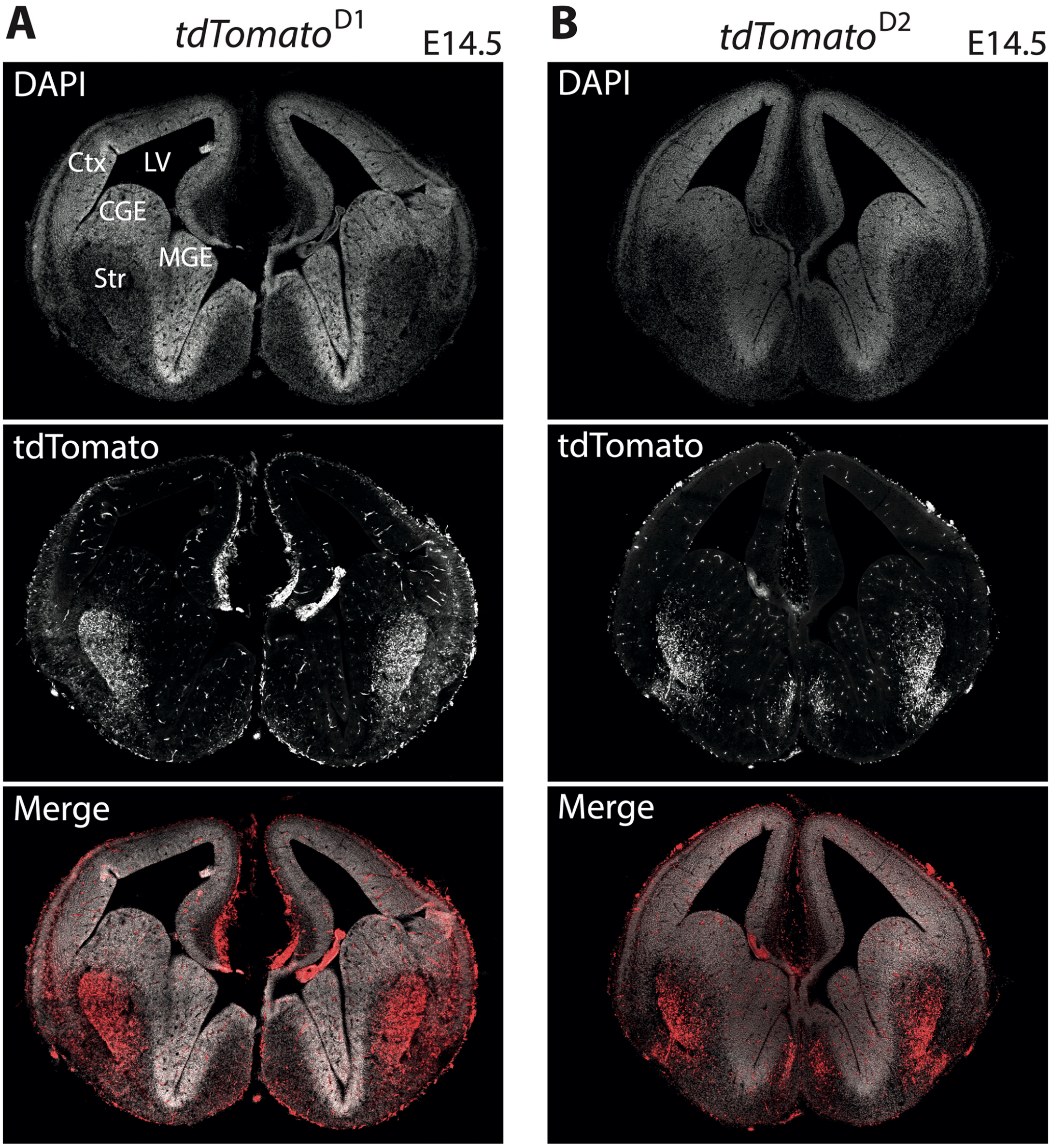

Fig. S10. Effective Cre recombination at El4.5 of Drd1-Cre and Drd2-Cre mice. DAPI and RFP (for tdTomato) co-staining on coronal sections in *tdTomato*D1 (Drdl-Cre x Ail 4; **A)** and *tdTomato*D2 (Drd2-Cre x Ail 4; **B)** mice at El4.5, showing effective recombination in the developing brain,with strong tdTomato expression in the developing striatum. The images were taken using a microscope 10X objective. LV: lateral ventricle; Ctx: cortex; CGE: caudal ganglionic eminence; MGE: medial ganglionic eminence; Str: striatum.

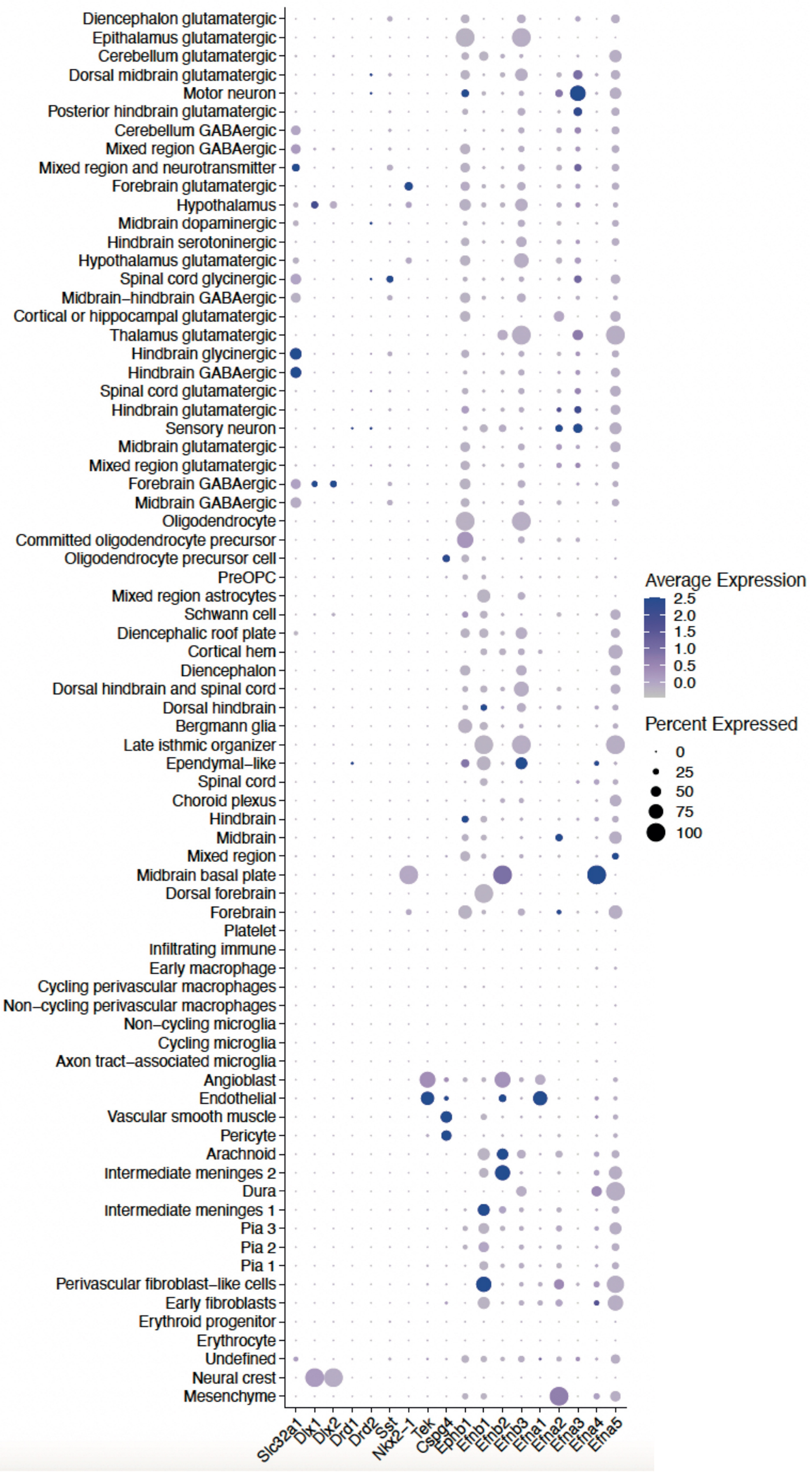
Diencephalon glutamatergic
Epithalamus glutamatergic
Cerebellum glutamatergic
Dorsal midbrain glutamatergic
Motor neuron
Posterior hindbrain glutamatergic
Cerebellum GABAergic
Mixed region GABAergic
Mixed region and neurotransmitter
Forebrain glutamatergic
Hypothalamus
Midbrain dopaminergic
Hindbrain serotoninergic
Hypothalamus glutamatergic
Spinal cord glycinergic
Midbrain–hindbrain GABAergic
Cortical or hippocampal glutamatergic
Thalamus glutamatergic
Hindbrain glycinergic
Hindbrain GABAergic
Spinal cord glutamatergic
Hindbrain glutamatergic
Sensory neuron
Midbrain glutamatergic
Mixed region glutamatergic
Forebrain GABAergic
Midbrain GABAergic
Oligodendrocyte
Committed oligodendrocyte precursor
Oligodendrocyte precursor cell
PreOPC
Mixed region astrocytes
Schwann cell
Diencephalic roof plate
Cortical hem
Diencephalon
Dorsal hindbrain and spinal cord
Dorsal hindbrain
Bergmann glia
Late isthmic organizer
Ependymal–like
Spinal cord
Choroid plexus
Hindbrain
Midbrain
Mixed region
Midbrain basal plate
Dorsal forebrain
Forebrain
Platelet
Infiltrating immune
Early macrophage
Cycling perivascular macrophages
Non–cycling perivascular macrophages
Non–cycling microglia
Cycling microglia
Axon tract–associated microglia
Angioblast
Endothelial
Vascular smooth muscle
Pericyte
Arachnoid
Intermediate meninges 2
Dura
Intermediate meninges 1
Pia 3
Pia 2
Pia 1
Perivascular fibroblast–like cells
Early fibroblasts
Erythroid progenitor
Erythrocyte
Undefined
Neural crest
Mesenchyme
Slc32a1
Dlx1
Dlx2
Drd1
Drd2
Sst
Nkx2–1
Tek
Cspg4
Ephb1
Efnb1
Efnb2
Efnb3
Efna1
Efna2
Efna3
Efna4
Efna5
Average Expression
2.5
2.0
1.5
1.0
0.5
0.0
Percent Expressed
0
25
50
75
100

Fig. S11. Gene expression at E14.5. Dot plot depicting the expression of our genes of interest. Gradient color corresponds to the expression level whereas dot size corresponds to the percentage of cells expressing the genes. Data was retrieved from mousebrain.org. *Slc32a1* gene codes for *Vgat; Tek* codes for *Tie2; Efnb1-3* genes code for ephrins B1-3; *Efna1-5* genes code for ephrins A 1-5.

Development • Supplementary information

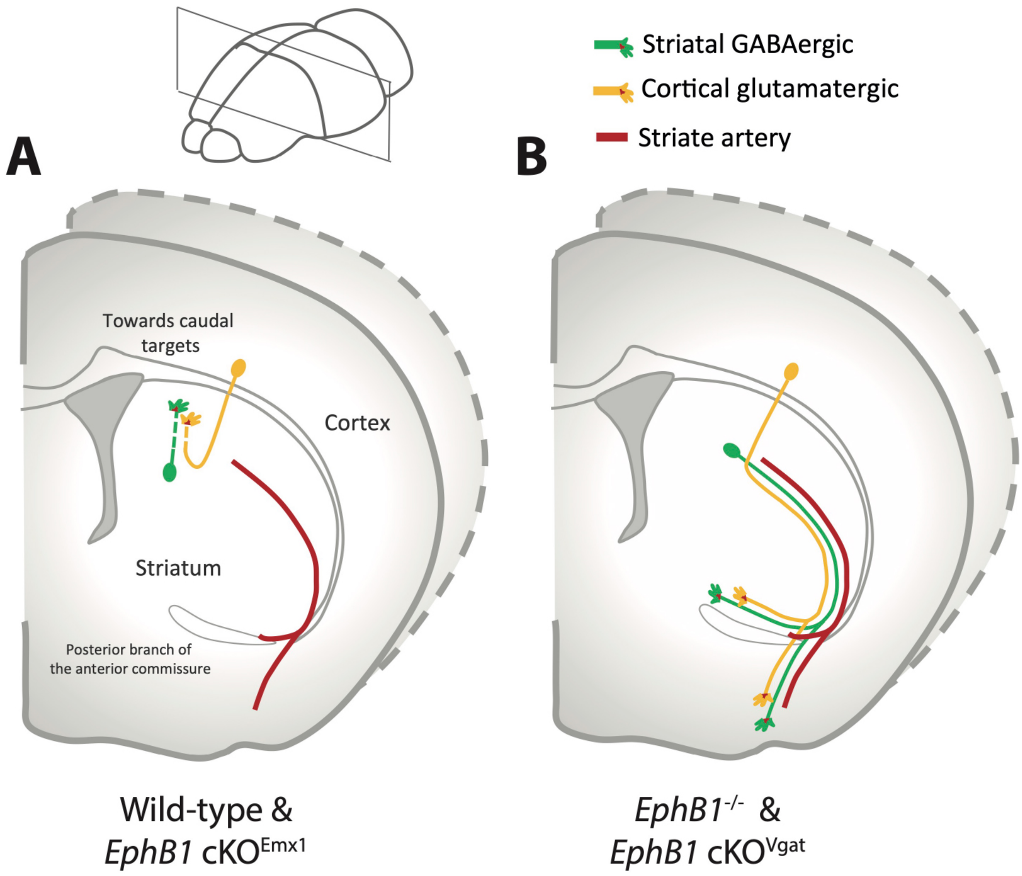

Fig. S12. Graphical summary of results. A. In Wild-Type mice: developing glutamatergic cortical axons navigating through the striatum follow developing striatal GABAergic axons to target their caudal brain regions. **B.** In absence of EphB1: 1. EphB1-expressing developing striatal GABAergic axons ectopically follow developing blood vessels, probably via a lack of vasculature ephrin-B-mediated repulsion. 2. Since cortical axons normally follow striatal axons in the striatum, misprojected GABAergic axons cause cortical axon misrouting through a cell non-autonomous mechanism.

Table S1. Percentage of mice presenting disorganised and misprojected fiber bundles for each genotype.

	Percentage of mice presenting disorganised and misprojected fiber bundles		
	descending in the dorsal striatum	descending from the external capsule and terminating near the brain floor	descending in the internal capsule
EphB1 $^{-/-}$	100%	100%	100%
EphB1 $^{\Delta Ex3/\Delta Ex3}$	100%	100%	100%
EphB1 cKO^{Emx}	0%	0%	0%
EphB1 cKO^{Vgat}	100%	100%	100%
EphB1 cKO^{D1}	0%	0%	0%
EphB1 cKO^{D2}	0%	0%	0%
EphB1 cKO^{Tie2}	0%	0%	0%
