# Supplementary material for: EphB1 controls long-range cortical axon guidance through a cell non-autonomous role in GABAergic cells
Source: Development. 2024 Feb 28;151(5):dev201439. doi: 10.1242/dev.201439 (PMC10946438; doi:10.1242/dev.201439)
Supplement: Supplementary file 1 [file develop-151-201439_review_history.pdf]

EphB1 controls long-range cortical axon guidance through a cell non-autonomous role in GABAergic cells

Ahlem Assali, George Chenaux, Jennifer Y. Cho, Stefano Berto, Nathan A. Ehrlich and Christopher W. Cowan

DOI: 10.1242/dev.201439

Editor: Debra Silver

Review timeline

Original submission:	15 November 2022
Editorial decision:	19 December 2022
First revision received:	20 July 2023
Editorial decision:	26 July 2023
Second revision received:	23 August 2023
Editorial decision:	2 October 2023
Third revision received:	24 January 2024
Accepted:	28 January 2024

Original submission

First decision letter

MS ID#: DEVELOP/2022/201439

MS TITLE: EphB1 controls proper long-range cortical axon guidance through a cell non-autonomous role in GABAergic cells

AUTHORS: Ahlem Assali, George Chenaux, and Christopher Cowan

I have now received all the referees' reports on the above manuscript, and have reached a decision. The referees' comments are appended below, or you can access them online: please go to BenchPress and click on the 'Manuscripts with Decisions' queue in the Author Area.

As you will see, the referees express considerable interest in your work, but have some significant criticisms and recommend a substantial revision of your manuscript before we can consider publication. You will note the specific concerns raised at the end of this letter. Several reviewers point out that to interpret negative phenotypes it is essential to better validate deletion of EphB1 (perhaps with RNAscope as used for Vgat neurons) including at embryonic stages. Additionally, they point out that quantification is needed throughout to rigorously support conclusions. Further, a systematic analysis of EphrinB expression patterns is needed to support and interpret the findings regarding cell specific functions.

If you are able to revise the manuscript along the lines suggested, I will be happy receive a revised version of the manuscript. Your revised paper will be re-reviewed by one or more of the original referees, and acceptance of your manuscript will depend on your addressing satisfactorily the reviewers' major concerns. Please also note that Development will normally permit only one round of major revision. If it would be helpful, you are welcome to contact us to discuss your revision in greater detail. Please send us a point-by-point response indicating your plans for addressing the referee’s comments, and we will look over this and provide further guidance.

© 2023. Published by The Company of Biologists under the terms of the Creative Commons Attribution License (https://creativecommons.org/licenses/by/4.0/).

Please attend to all of the reviewers' comments and ensure that you clearly highlight all changes made in the revised manuscript. Please avoid using 'Tracked changes' in Word files as these are lost in PDF conversion. I should be grateful if you would also provide a point-by-point response detailing how you have dealt with the points raised by the reviewers in the 'Response to Reviewers' box. If you do not agree with any of their criticisms or suggestions please explain clearly why this is so.

Reviewer 1

Advance summary and potential significance to field

Defects in cortical axons in EphB1 mutant mice is likely not due to cell-autonomous mechanisms. The authors identified that some population(s) of GABAergic neurons are the primary cause of non-cell autonomous defects of cortical axon development. This result has a potential to enhance the understanding of the role of axon guidance receptors in brain development.

Comments for the author

In this manuscript, Assali et. al. sought to determine the cellular basis of EphB1 function in brain development. Previous studies by the authors' lab and others found that germ-line deletion of the EphB1 gene results in axon development defects in the brain. In order to identity the responsible cell populations, they developed floxed mutant mice and used a wide variety of Cre lines to conditionally knock out EphB1 so they can find which conditional mutants phenocopy the germ-line mutants. First, they confirmed multiple guidance defects at P0 including disorganized axons and ectopic axon bundles in the internal capsule, and ectopic axon projections descending from the external capsules. The authors then looked at thalamocortical axon defects, which they authors previously found in EphB1/B2 double mutants. However, in single EphB1 knockout mice, these axons remained intact, indicating that the cortical axon defects are not due to the aberrant thalamocortical axons. In addition, they corrected their previous paper by showing that EphB1 is in fact not detected in the thalamus, although it is expressed in the habenula. Next, deleting EphB1 in cortical excitatory neurons with the Emx1-Cre driver did not result in cortical axon defects, suggesting that these defects are non-cell autonomous. In contrast, drivers for GABAerginc neurons (Vgat-Cre and Dlx1/2-Cre) did cause similar defects in cortical axons, and genetic and viral axon labeling of GABAergic and cortical excitatory neurons suggests that ectopic GABAergic axons (not from the cortex) co-fasciculate with the misguided cortical axons. Further narrowing down the subpopulations of GABAergic neurons with Nkx2.1-Cre, SST-Cre Drdr1-Cre or Drd2-Cre drivers did not cause cortical axons defects, nor drivers specific to endothelial cells or mural and oligodendrocyte precursor cells. The results are generally solid and support the authors' conclusions with some exceptions described below. It is of significance that the study suggests that cortical axon defects are not due to the lack of EphB1 in the originating neurons, but due to the non-cell autonomous defects in GABAergic neurons.

However, they did not identify specific population(s) of GABAergic neurons responsible for such a role of EphB1 despite the use of multiple Cre drivers to conditionally knock out EphB1, which reduces the impact of the work. It is possible that some Cre drivers did not cause significant deletion of EphB1 at the right stage of development, but the authors show no validation to rule out such possibilities. It would also be much more informative if the authors show data at early embryonic stages when the axons are still growing, as they did in their own Robichaux et al. paper in 2014; in the current study, the only data shown are P0 or adult stages. These data inform presumably the final outcome of the aberrancy, but not the process that lead to the phenotypes, falling short of providing insights into the mechanisms.

Below are some additional issues:
Relationship between Fig.2A-D and Fig.2E-F in terms of locations is not clear.
Data shown in Suppl. Fig.7 is unclear. Do the authors suggest that there is any difference between cKO and control brains regarding the co-mingling of cortical and striatal axons projecting towards the globus pallidus?

Reviewer 2

© 2023. Published by The Company of Biologists under the terms of the Creative Commons Attribution License (https://creativecommons.org/licenses/by/4.0/).

Advance summary and potential significance to field

This manuscript by Assali et al. investigates the role of the receptor EphB1 in controling axon guidance in the developing mouse cortex. Previous studies have shown that EphB1 knock out mice have defects in the guidance of cortical axons, however, how EphB1 controlled these axons and more precisely in which cell compartment was it required remained unknown. The authors tackled this question using mouse genetics with a panel of Cre lines together with a new EphB1 conditional allele. They show that EphB1 is required in GABAergic neurons but not in glutamatergic cortical neurons to ensure axon guidance uncovering a non autonomous mode of action. In addition, they used Cre lines specific for oligodendrocytes precursors and for endothelial cells and observed that conditional mutants did not exhibit axon guidance defects, indicating no requirement of EphB1 in these cell compartments.
This is an interesting mouse genetic study with a number of mouse lines (including a new conditional allele for EphB1) that tease out the mode of action of EphB1 in the developing brain.

Comments for the author

The data is mostly descriptive and the study lacks essential data including quantification. Further, the added value of some of the Cre lines is not completely clear.

Specific comments :
-None of the reported phenotypes are quantified in any way. The authors should include some quantifications to give an idea of the robustness of the phenotypes described.

-The expression of EphB1 in the different cell compartments should be shown, either using the LacZ line or by In situ hybridization.

-Similarly, the loss of EphB1 expression in these compartments following Cre excision should be shown on tissue sections.

-The added value of using Cspg4-Cre or Tie2-Cre is not completely clear, this part seems a bit distinct from the rest of the study. Further, the fact that axon bundles follow vasculature in the EphB1 cKO could be due to a role of blood vessels as non specific barriers that wandering axons « hit » and follow. In my opinion it does not necessarily imply that EphB1 plays an active role in promoting avoidance of blood vessels. More data is needed to fully support this notion.

- in the discussion the authors focus on endothelial-specific ephrinB2 but they do not discuss the role of ephrinB2 (or other ephrins) as ligands expressed in glutamatergic cortical neurons. Do ephrin mutants (conditional and /or constitutive) exhibit axon guidance defects similar to EphB1 ? This point should be added in the discussion.

<u>Reviewer 3</u>

Advance summary and potential significance to field

In general, the Eph family is the largest family of tyrosine kinase receptors in the brain and plays fundamental roles in virtually all neuro-developmental processes. Due to their complex biology (multiple overlapping expression patterns and the potential for bi-directional signalling) makes this family a challenge to study. Therefore the work in this manuscript is highly appreciated in which a conditional KO is generated and with a multitude of different Cre lines investigated.

Defects in a knockout of the guidance receptor EphB1 have been noted but the mechanisms behind remained unknown. The approaches taken by the authors are a good way forward to elucidate these.

Comments for the author

© 2023. Published by The Company of Biologists under the terms of the Creative Commons Attribution License (https://creativecommons.org/licenses/by/4.0/).

A full KO of the receptor EphB1 results in axon guidance defects of axons from cells in the deeper layers of the somatosensory cortex; however the cell types affected and the precise role/mechanism of EphB1 in this processes deduced from the observed defects had not be described in further detail so far. Since members of the Eph family signal/function bi-directionally, multiple mechanisms to explain these defects appear possible.

The authors generated a novel floxed EphB1 allele and bred this with a massive battery of different Cre lines, and did a careful and detailed analysis to gain further insight into this interesting question.

Conditional KO of EphB1 in excitatory neurons of the deep layers of the somatosensory cortex did not lead to guidance defects, but a cKO of EphB1 in GABAergic neurons of the striatum shows guidance defects mimicking the full KO. Thus, in this particular cKO defects of axons of excitatory neurons from the somatosensory cortex now were observed. This suggests a non-cell autonomous defect from a cKO of EphB1 in striatal GABAergic but not cortical GABAergic neurons onto excitatory axons/neurons from the somatosensory cortex, i.e. some disturbed fasciculation is discussed.

Surprisingly, a cKO of EphB1 in dopaminergic neurons of the striatum did not lead to these guidance defects.
Investigation of a possible role of EphB1 in endothelial cells and other cell types of the vasculature – approached using further Cre lines did not elucidate the mechanisms of disturbed axon fascicle formation.

Comments
Overall, the topic, the manuscript, and the large number of cre lines used appear quite complex, making it wishful to include some schemes of the results.

I find it surprising that very little attention is being paid to the role of ephrinBs in this manuscript.

Occasionally but more spontaneously, an apparent (co-) expression on axon tracts and else structures with EphBs is noted, but overall a systemic inclusion of expression patterns and their possible role in explaining the EphB1 defects is lacking, but very wishful to see, that is some key expression data for ephrinBs appear necessary to be included in this manuscript.

It is claimed that little is known about brain vasculature and CNS axon guidance, in fact there is quite an extensive literature which should be referred to properly.

First revision

Author response to reviewers' comments

Reviewer 1 Advance Summary and Potential Significance to Field:
Defects in cortical axons in EphB1 mutant mice is likely not due to cell- autonomous mechanisms. The authors identified that some population(s) of GABAergic neurons are the primary cause of non-cell autonomous defects of cortical axon development. This result has a potential to enhance the understanding of the role of axon guidance receptors in brain development.

Reviewer 1 Comments for the Author:
In this manuscript, Assali et. al. sought to determine the cellular basis of EphB1 function in brain development. Previous studies by the authors' lab and others found that germ-line deletion of the EphB1 gene results in axon development defects in the brain. In order to identity the responsible cell populations, they developed floxed mutant mice and used a wide variety of Cre lines to conditionally knock out EphB1 so they can find which conditional mutants phenocopy the germ-line mutants. First, they confirmed multiple guidance defects at P0 including disorganized axons and ectopic axon bundles in the internal capsule, and ectopic axon projections descending from the external capsules. The authors then looked at thalamocortical axon defects, which they authors

© 2023. Published by The Company of Biologists under the terms of the Creative Commons Attribution License (https://creativecommons.org/licenses/by/4.0/).

previously found in EphB1/B2 double mutants. However, in single EphB1 knockout mice, these axons remained intact, indicating that the cortical axon defects are not due to the aberrant thalamocortical axons. In addition, they corrected their previous paper by showing that EphB1 is in fact not detected in the thalamus, although it is expressed in the habenula. Next, deleting EphB1 in cortical excitatory neurons with the Emx1-Cre driver did not result in cortical axon defects, suggesting that these defects are non-cell autonomous. In contrast, drivers for GABAergic neurons (Vgat-Cre and Dlx1/2-Cre) did cause similar defects in cortical axons, and genetic and viral axon labeling of GABAergic and cortical excitatory neurons suggests that ectopic GABAergic axons (not from the cortex) co-fasciculate with the misguided cortical axons. Further narrowing down the subpopulations of GABAergic neurons with Nkx2.1-Cre, SST-Cre, Drdr1-Cre or Drd2-Cre drivers did not cause cortical axons defects, nor drivers specific to endothelial cells or mural and oligodendrocyte precursor cells. The results are generally solid and support the authors' conclusions with some exceptions described below. It is of significance that the study suggests that cortical axon defects are not due to the lack of EphB1 in the originating neurons, but due to the non-cell autonomous defects in GABAergic neurons. However, they did not identify specific population(s) of GABAergic neurons responsible for such a role of EphB1 despite the use of multiple Cre drivers to conditionally knock out EphB1, which reduces the impact of the work. **It is possible that some Cre drivers did not cause significant deletion of EphB1 at the right stage of development, but the authors show no validation to rule out such possibilities.**

We found misguided long-range striatal GABAergic projections, but EphB1 deletion from Drd1+ and Drd2+ cells did not phenocopy the *EphB1*$^{-/-}$ mice. We totally agree with reviewer 1 on the fact that Drd1-Cre and Drd2-Cre might not recombine early enough to affect the axon guidance process we're interested in, therefore preventing us from drawing a clear conclusion. To address this issue, we crossed the Drd1-Cre and Drd2-Cre mice with Ai14 reporter mice and found clear recombination at E14.5. This finding is now included in the Suppl. Fig. 10 in our revised manuscript.

The other Cre lines that we used have been extensively characterized in other publications [1-4]. In addition, we collaborated with Stefano Berto, a bioinformatician, to analyze the published single-cell RNAseq dataset of whole embryonic mouse brains [5] and confirmed expression of Tek (Tie-Cre), Cspg4 (Cspg4-Cre), Sst (Sst-Cre) at E14.5 in the appropriate cell types. The results of this analysis have been added to our revised manuscript in Suppl. Fig. 11.

It would also be much more informative if the authors show data at early embryonic stages when the axons are still growing, as they did in their own Robichaux et al. paper in 2014; in the current study, the only data shown are P0 or adult stages. These data inform presumably the final outcome of the aberrancy, but not the process that leads to the phenotypes, falling short of providing insights into the mechanisms.

We agree with reviewer 1. We tried to visualize the GABAergic axons at earlier ages, using the EphB1 cKO^{Vgat}; tdTomato^{Vgat} to label all GABAergic axons with tdTomato in the context of the EphB1 deletion in Vgat+ cells. We found misguided GABAergic axons among the L1CAM+ ectopic axons at E15.5, suggesting that the GABAergic axon guidance deficits emerge at least as early as the cortical ones. Indeed, in our previous work, we found cortical misguided axons at E16.5 [6]. We added these data to our revised manuscript in Suppl. Fig. 9. However, since GABAergic neurons have already migrated and invaded many brain regions and mainly the developing striatum at this early embryonic age, GABAergic axons were not discernible from the GABAergic cells, preventing us to further analyze the process that leads to the axon guidance deficits.

Below are some additional issues:

Relationship between Fig.2A-D and Fig.2E-F in terms of locations is not clear.

We now added annotations on the images in Fig. 2E-F to clarify the locations in the ventral part of the brain (ec: external capsule; pac: posterior branch of the anterior commissure; str: striatum; hyp: hypothalamus; MAN: medial amygdalar nucleus).

Data shown in Suppl. Fig.7 is unclear. Do the authors suggest that there is any difference between cKO and control brains regarding the co-mingling of cortical and striatal axons projecting towards the globus pallidus?

We did not observe differences between the cKOs and WTs mice regarding this co-mingling. To overcome this confusion, we removed the cKOs images (now Suppl. Fig.8), as the aim of this supplementary figure was to show that in WT mice, we noticed co-mingling of cortical and striatal axons projecting towards the globus pallidus, suggesting that cortical and striatal axons follow each

© 2023. Published by The Company of Biologists under the terms of the Creative Commons Attribution License (https://creativecommons.org/licenses/by/4.0/).

other, and suggesting that misguided striatal axons could consequently misguide cortical axons that co-mingle with them.

Reviewer 2 Advance Summary and Potential Significance to Field: This manuscript by Assali et al. investigates the role of the receptor EphB1 in controlling axon guidance in the developing mouse cortex. Previous studies have shown that EphB1 knock out mice have defects in the guidance of cortical axons, however, how EphB1 controlled these axons and more precisely in which cell compartment was it required remained unknown. The authors tackled this question using mouse genetics with a panel of Cre lines together with a new EphB1 conditional allele. They show that EphB1 is required in GABAergic neurons but not in glutamatergic cortical neurons to ensure axon guidance uncovering a non-autonomous mode of action. In addition, they used Cre lines specific for oligodendrocytes precursors and for endothelial cells and observed that conditional mutants did not exhibit axon guidance defects, indicating no requirement of EphB1 in these cell compartments. This is an interesting mouse genetic study with a number of mouse lines (including a new conditional allele for EphB1) that tease out the mode of action of EphB1 in the developing brain.

Reviewer 2 Comments for the Author:

The data is mostly descriptive, and the study lacks essential data including quantification. Further, the added value of some of the Cre lines is not completely clear.

Specific comments:
-None of the reported phenotypes are quantified in any way. The authors should include some quantifications to give an idea of the robustness of the phenotypes described.
We thank reviewer 2 for his comment. The reason why we didn't include quantifications is that the phenotype is 100% penetrant. Every single *EphB1*$^{-/-}$ mouse, *EphB1*$^{\Delta Ex3/\Delta Ex3}$ mouse, *EphB1* cKO^{Vgat} mouse and *EphB1* cKO$^{dlx1/2}$ mouse clearly displayed the axon guidance deficits described in Figure 1. The phenotype is super robust and 100% consistent.

-The expression of EphB1 in the different cell compartments should be shown, either using the LacZ line or by in situ hybridization.
We reported relevant EphB1 expression in the mouse embryo brain in our previous article [6]. However, we agree with reviewer 2 that it was important to assess EphB1 expression at E14.5 in the different cell types of the mouse embryo, right before the axon guidance process we are interested in. For that, we collaborated with Stefano Berto, a bioinformatician, to analyze the published single cell RNAseq dataset of whole embryonic mouse brains [5]. We found that EphB1 is strongly expressed in the expected cell types, such as forebrain GABAergic and glutamatergic cells, or oligodendrocytes. Interestingly, using this dataset, we discovered that EphB1 is also expressed in angioblasts, endothelial cells precursors. However, our work showed that EphB1 deletion from endothelial cells did not phenocopy the defects observed in *EphB1*$^{-/-}$ mice. We now added this detailed analysis of EphB1 expression in Suppl. Fig.11.

-Similarly, the loss of EphB1 expression in these compartments following Cre excision should be shown on tissue sections.
Reviewer 1 had a similar request. We found misguided long-range striatal GABAergic projections, but EphB1 deletion from Drd1+ and Drd2+ cells did not phenocopy the EphB1$^{-/-}$ mice. However, Drd1-Cre and Drd2-Cre might not recombine early enough to affect the axon guidance process we're interested in. To address this issue, we crossed the Drd1-Cre and Drd2-Cre mice with Ai14 reporter mice and found clear recombination at E14.5. This finding is now included in the Suppl. Fig. 10 in our revised manuscript.
The other Cre lines that we used have been extensively characterized in other publications [1-4]. In addition, we collaborated with Stefano Berto, a bioinformatician, to analyze the published single cell RNAseq dataset of whole embryonic mouse brains [5] and confirmed expression of Tek (Tie-Cre), Cspg4 (Cspg4-Cre), Sst (Sst-Cre) at E14.5. The results of this analysis have been added to our revised manuscript as Suppl. Fig. 11.

-The added value of using Cspg4-Cre or Tie2-Cre is not completely clear, this part seems a bit distinct from the rest of the study.

© 2023. Published by The Company of Biologists under the terms of the Creative Commons Attribution License (https://creativecommons.org/licenses/by/4.0/).

EphB1 deletion using the Dlx1/2- and Vgat-Cre lines phenocopied the defects found in the *EphB1*$^{-/-}$ mice. This finding strongly suggested a role of EphB1 in GABAergic neurons. However, dlx and Vgat are not only expressed in GABAergic neurons early on, but they're also expressed in endothelial cells and dlx2 is expressed in a pool of progenitors giving rise to oligodendrocyte precursor cells (OPCs), known to migrate along blood vessels. EphB1 is expressed in OPCS, and using the single cell RNAseq dataset from E14.5 embryos, we found that EphB1 is also expressed in angioblasts. This suggests that using the dlx and Vgat-Cre lines, EphB1 is likely deleted from endothelial cells and OPCs. Therefore, EphB1 could have been playing a role in these latter cells, to repulse ephrinB-expressing axons. To rule out the possibility of a function of EphB1 in these endothelial or OPCs cells, we used the Tie2-Cre line to delete EphB1 from endothelial cells and NG2-Cre line to delete EphB1 from OPCs and mural cells. We didn't find any deficits. This analysis seemed important to us for understanding the mechanism underlying our phenotype, which is why we've included it in the manuscript

Further, the fact that axon bundles follow vasculature in the EphB1 cKO could be due to a role of blood vessels as non-specific barriers that wandering axons « hit » and follow. In my opinion it does not necessarily imply that EphB1 plays an active role in promoting avoidance of blood vessels. More data is needed to fully support this notion.
We agree with reviewer 2 that we can't exclude that possible explanation of the phenotype. We've added a sentence to the revised discussion acknowledging the possibility that the blood vessel phenotype is not related to active EphB1-mediated avoidance of blood vessels. Future studies will be needed to better understand the axon/blood vessel phenotype in the EphB1 mutants, as the reviewer notes.

-In the discussion the authors focus on endothelial-specific ephrinB2, but they do not discuss the role of ephrinB2 (or other ephrins) as ligands expressed in glutamatergic cortical neurons. Do ephrin mutants (conditional and /or constitutive) exhibit axon guidance defects similar to EphB1? This point should be added in the discussion.
We agree with the reviewer that this is an important point to add to the discussion. We now added the following paragraph to our discussion in the revised manuscript:
"EphB1 mediates many of its biological functions through bidirectional signaling induced by contact-mediated binding to ephrins. EphrinB2 is strongly expressed in the developing cortex and striatum [2], and we previously showed that conditional deletion of ephrinB2 in Nestin-Cre mice, where recombination includes cortical glutamatergic neurons, did not phenocopy the axon guidance deficits found in EphB1$^{-/-}$ mice [2]. Conditional deletion of ephrinB2 using the FoxG1-Cre line produced thalamo-cortical axon guidance deficits similar to those observed in EphB1$^{-/-}$;EphB2$^{-/-}$ mice, but did not phenocopy the cortical axon guidance deficits found in EphB1$^{-/-}$ mice and in EphB1$^{-/-}$;EphB2$^{-/-}$ mice. EphrinB3, also expressed in the cortex and hippocampus, has been shown to control corticospinal tract axon guidance [44], but future studies will be needed to assess its possible role in the EphB1 cKO^{Vgat} phenotype."

Reviewer 3 Advance Summary and Potential Significance to Field:
In general, the Eph family is the largest family of tyrosine kinase receptors in the brain and plays fundamental roles in virtually all neuro-developmental processes. Due to their complex biology (multiple overlapping expression patterns and the potential for bi-directional signaling makes this family a challenge to study. Therefore, the work in this manuscript is highly appreciated in which a conditional KO is generated and with a multitude of different Cre lines investigated.

Defects in a knockout of the guidance receptor EphB1 have been noted but the mechanisms behind remained unknown. The approaches taken by the authors are a good way forward to elucidate these.

Reviewer 3 Comments for the Author:
A full KO of the receptor EphB1 results in axon guidance defects of axons from cells in the deeper layers of the somatosensory cortex; however, the cell types affected and the precise role/mechanism of EphB1 in this processes deduced from the observed defects had not be described in further detail so far. Since members of the Eph family signal/function bi-directionally, multiple mechanisms to explain these defects appear possible.

© 2023. Published by The Company of Biologists under the terms of the Creative Commons Attribution License (https://creativecommons.org/licenses/by/4.0/).

The authors generated a novel floxed EphB1 allele and bred this with a massive battery of different Cre lines, and did a careful and detailed analysis to gain further insight into this interesting question.

Conditional KO of EphB1 in excitatory neurons of the deep layers of the somatosensory cortex did not lead to guidance defects, but a cKO of EphB1 in GABAergic neurons of the striatum shows guidance defects mimicking the full KO. Thus, in this particular cKO defects of axons of excitatory neurons from the somatosensory cortex now were observed. This suggests a non-cell autonomous defect from a cKO of EphB1 in striatal GABAergic but not cortical GABAergic neurons onto excitatory axons/neurons from the somatosensory cortex, i.e. some disturbed fasciculation is discussed.

Surprisingly, a cKO of EphB1 in dopaminergic neurons of the striatum did not lead to these guidance defects.

Investigation of a possible role of EphB1 in endothelial cells and other cell types of the vasculature - approached using further Cre lines did not elucidate the mechanisms of disturbed axon fascicle formation.

Comments

Overall, the topic, the manuscript, and the large number of cre lines used appear quite complex, making it wishful to include some schemes of the results.
This is a good suggestion. We now included a scheme that summarizes our finding in Supplemental Figure 12.

I find it surprising that very little attention is being paid to the role of ephrinBs in this manuscript. Occasionally but more spontaneously, an apparent (co-) expression on axon tracts and else structures with EphBs is noted, but overall a systemic inclusion of expression patterns and their possible role in explaining the EphB1 defects is lacking, but very wishful to see, that is **some key expression data for ephrinBs appear necessary to be included in this manuscript**.
We previously reported ephrinB2 spatial expression at E13 [6], using the ephrinB2-LacZ mice. EphrinB2 is strongly expressed in the developing cortex and developing striatum. We now added this information to our revised manuscript. There are 3 ephrinBs expressed in the brain, ephrinB 1-3. None of them have good antibodies. To precisely assess ephrinB (and ephrinA) expression at E14.5 in the different cell types of the mouse embryo, right before the axon guidance process we are interested in, we analyzed the published single cell RNAseq dataset of embryonic mouse brains [5], now included in the Suppl. Fig. 11. In our first submission, we discussed a potential role of ephrinBs in developing vasculature. In addition to that, we now added another paragraph in the discussion about ephrinBs' potential role in our phenotype.

It is claimed that little is known about brain vasculature and CNS axon guidance, in fact there is quite an extensive literature which should be referred to properly.
We now removed this inappropriate statement, but we've kept the articles we cited in the discussion about this general topic.

1. Liang, H., S. Hippenmeyer, and H.T. Ghashghaei, *A Nestin-cre transgenic mouse is insufficient for recombination in early embryonic neural progenitors*. Biol Open, 2012. **1**(12): p. 1200-3.
2. Potter, G.B., et al., *Generation of Cre-transgenic mice using Dlx1/Dlx2 enhancers and their characterization in GABAergic interneurons*. Mol Cell Neurosci, 2009. **40**(2): p. 167-86.
3. Saunders, A., K.W. Huang, and B.L. Sabatini, *Globus Pallidus Externus Neurons Expressing parvalbumin Interconnect the Subthalamic Nucleus and Striatal Interneurons*. PLoS One, 2016. **11**(2): p. e0149798.
4. Kisanuki, Y.Y., et al., *Tie2-Cre transgenic mice: a new model for endothelial cell-lineage analysis in vivo*. Dev Biol, 2001. **230**(2): p. 230-42.
5. La Manno, G., et al., *Molecular architecture of the developing mouse brain*. Nature, 2021. **596**(7870): p. 92-96.
6. Robichaux, M.A., et al., *EphB receptor forward signaling regulates area-specific reciprocal thalamic and cortical axon pathfinding*. Proc Natl Acad Sci U S A, 2014. **111**(6): p. 2188-93.

© 2023. Published by The Company of Biologists under the terms of the Creative Commons Attribution License (https://creativecommons.org/licenses/by/4.0/).

Second decision letter

MS ID#: DEVELOP/2022/201439

MS TITLE: EphB1 controls proper long-range cortical axon guidance through a cell non-autonomous role in GABAergic cells

AUTHORS: Ahlem Assali, George Chenaux, Jennifer Y Cho, Stefano Berto, Nathan A Ehrlich, and Christopher Cowan

Thank you for sending your revised manuscript. I have carefully read the response and new draft. I am concerned there are several remaining major concerns which have not been adequately addressed (as outlined in my original letter and noted by several reviewers). I am returning this to you to tackle these issues now, before I send this to reviewers; Development only allows one round of major revisions this may avoid having to reject after review.

Specific concerns which remain are as follows:

1) Two reviewers asked for validated analysis of EphB1 expression (using your RNAscope tools or antibody) in the Cre experiments with negative results. Ai14 is a genetic reporter for recombination but this is not the same as directly monitoring the target gene.

2) Reviewer 2 raised the concern that quantifications are needed throughout the paper. Even if phenotypes are 100% penetrant, quantifications are critical for rigorously reporting strength of phenotypes as well as demonstrating data is collected across many biological samples.

Please let me know if you have questions and I look forward to receiving your revised manuscript.

Your revised paper will be re-reviewed by one or more of the original referees, and acceptance of your manuscript will depend on your addressing satisfactorily the reviewers' major concerns. Please also note that Development will normally permit only one round of major revision. If it would be helpful, you are welcome to contact us to discuss your revision in greater detail. Please send us a point-by-point response indicating your plans for addressing the referees' comments, and we will look over this and provide further guidance.

Please attend to all of the reviewers' comments and ensure that you clearly highlight all changes made in the revised manuscript. Please avoid using 'Tracked changes' in Word files as these are lost in PDF conversion. I should be grateful if you would also provide a point-by-point response detailing how you have dealt with the points raised by the reviewers in the 'Response to Reviewers' box. If you do not agree with any of their criticisms or suggestions please explain clearly why this is so.

Second revision

Author response to reviewers' comments

Reviewer 1 Advance Summary and Potential Significance to Field:
Defects in cortical axons in EphB1 mutant mice is likely not due to cell- autonomous mechanisms. The authors identified that some population(s) of GABAergic neurons are the primary cause of non-cell autonomous defects of cortical axon development. This result has a potential to enhance the understanding of the role of axon guidance receptors in brain development.

Reviewer 1 Comments for the Author:
In this manuscript, Assali et. al. sought to determine the cellular basis of EphB1 function in brain development. Previous studies by the authors' lab and others found that germ-line deletion of the

© 2023. Published by The Company of Biologists under the terms of the Creative Commons Attribution License (https://creativecommons.org/licenses/by/4.0/).

EphB1 gene results in axon development defects in the brain. In order to identity the responsible cell populations, they developed floxed mutant mice and used a wide variety of Cre lines to conditionally knock out EphB1 so they can find which conditional mutants phenocopy the germ-line mutants. First, they confirmed multiple guidance defects at P0 including disorganized axons and ectopic axon bundles in the internal capsule, and ectopic axon projections descending from the external capsules. The authors then looked at thalamocortical axon defects, which they authors previously found in EphB1/B2 double mutants. However, in single EphB1 knockout mice, these axons remained intact, indicating that the cortical axon defects are not due to the aberrant thalamocortical axons. In addition, they corrected their previous paper by showing that EphB1 is in fact not detected in the thalamus, although it is expressed in the habenula. Next, deleting EphB1 in cortical excitatory neurons with the Emx1-Cre driver did not result in cortical axon defects, suggesting that these defects are non-cell autonomous. In contrast, drivers for GABAergic neurons (Vgat-Cre and Dlx1/2-Cre) did cause similar defects in cortical axons, and genetic and viral axon labeling of GABAergic and cortical excitatory neurons suggests that ectopic GABAergic axons (not from the cortex) co-fasciculate with the misguided cortical axons. Further narrowing down the subpopulations of GABAergic neurons with Nkx2.1-Cre, SST-Cre, Drdr1-Cre or Drd2-Cre drivers did not cause cortical axons defects, nor drivers specific to endothelial cells or mural and oligodendrocyte precursor cells. The results are generally solid and support the authors' conclusions with some exceptions described below. It is of significance that the study suggests that cortical axon defects are not due to the lack of EphB1 in the originating neurons, but due to the non-cell autonomous defects in GABAergic neurons. However, they did not identify specific population(s) of GABAergic neurons responsible for such a role of EphB1 despite the use of multiple Cre drivers to conditionally knock out EphB1, which reduces the impact of the work. **It is possible that some Cre drivers did not cause significant deletion of EphB1 at the right stage of development, but the authors show no validation to rule out such possibilities.**
We found misguided long-range striatal GABAergic projections, but EphB1 deletion from Drd1+ and Drd2+ cells did not phenocopy the *EphB1*$^{-/-}$ mice. We totally agree with reviewer 1 on the fact that Drd1-Cre and Drd2-Cre might not recombine early enough to affect the axon guidance process we're interested in, therefore preventing us from drawing a clear conclusion. Due to loss of funding for this study since a while and to Dr. Assali's departure to France for a new job in research in July 2022, it was not possible for us to confirm EphB1 recombination using the different cre lines. We were forced to stop the numerous and expensive crosses of this project and to cryopreserve the sperm from the EphB1 floxed mice. However, to address this issue the best way we can, we crossed the Drd1-Cre and Drd2-Cre mice with Ai14 reporter mice and found clear recombination at E14.5. This finding is now included in the Suppl. Fig. 10 in our revised manuscript. The Drd1-Cre and Drd2-Cre were the most important cre lines, since we found misguided long-range GABAergic projections from the striatum. The other Cre lines that we used have been extensively characterized in other publications [1-4]. In addition, we collaborated with Stefano Berto, a bioinformatician, to analyze the published single-cell RNAseq dataset of whole embryonic mouse brains [5] and confirmed expression of Tek (Tie-Cre), Cspg4 (Cspg4-Cre), Sst (Sst-Cre) at E14.5 in the appropriate cell types. The results of this analysis have been added to our revised manuscript in Suppl. Fig. 11.

It would also be much more informative if the authors show data at early embryonic stages when the axons are still growing, as they did in their own Robichaux et al. paper in 2014; in the current study, the only data shown are P0 or adult stages. These data inform presumably the final outcome of the aberrancy, but not the process that leads to the phenotypes, falling short of providing insights into the mechanisms.
We agree with reviewer 1. We already tried to visualize the GABAergic axons at earlier ages, using the EphB1 cKO^{Vgat}; tdTomato^{Vgat} to label all GABAergic axons with tdTomato in the context of the EphB1 deletion in Vgat+ cells, but we did not initially include these data in the manuscript. We found misguided GABAergic axons among the L1CAM+ ectopic axons at E15.5, suggesting that the GABAergic axon guidance deficits emerge at least as early as the cortical ones. Indeed, in our previous work, we found cortical misguided axons at E16.5 [6]. We added these data to our revised manuscript in Suppl. Fig. 9. However, since GABAergic neurons have already migrated and invaded many brain regions and mainly the developing striatum at this early embryonic age, GABAergic axons were not discernible from the GABAergic cells, preventing us to further analyze the process that leads to the axon guidance deficits.

Below are some additional issues:

© 2023. Published by The Company of Biologists under the terms of the Creative Commons Attribution License (https://creativecommons.org/licenses/by/4.0/).

Relationship between Fig.2A-D and Fig.2E-F in terms of locations is not clear.
We now added annotations on the images in Fig. 2E-F to clarify the locations in the ventral part of the brain (ec: external capsule; pac: posterior branch of the anterior commissure; str: striatum; hyp: hypothalamus; MAN: medial amygdalar nucleus).
Data shown in Suppl. Fig.7 is unclear. Do the authors suggest that there is any difference between cKO and control brains regarding the co-mingling of cortical and striatal axons projecting towards the globus pallidus?
We did not observe differences between the cKOs and WTs mice regarding this co-mingling. To overcome this confusion, we removed the cKOs images (now Suppl. Fig.8), as the aim of this supplementary figure was to show that in WT mice, we noticed co-mingling of cortical and striatal axons projecting towards the globus pallidus, suggesting that cortical and striatal axons follow each other, and suggesting that misguided striatal axons could consequently misguide cortical axons that co-mingle with them.

Reviewer 2 Advance Summary and Potential Significance to Field: This manuscript by Assali et al. investigates the role of the receptor EphB1 in controlling axon guidance in the developing mouse cortex. Previous studies have shown that EphB1 knock out mice have defects in the guidance of cortical axons, however, how EphB1 controlled these axons and more precisely in which cell compartment was it required remained unknown. The authors tackled this question using mouse genetics with a panel of Cre lines together with a new EphB1 conditional allele. They show that EphB1 is required in GABAergic neurons but not in glutamatergic cortical neurons to ensure axon guidance uncovering a non-autonomous mode of action. In addition, they used Cre lines specific for oligodendrocytes precursors and for endothelial cells and observed that conditional mutants did not exhibit axon guidance defects, indicating no requirement of EphB1 in these cell compartments. This is an interesting mouse genetic study with a number of mouse lines (including a new conditional allele for EphB1) that tease out the mode of action of EphB1 in the developing brain.

Reviewer 2 Comments for the Author:
The data is mostly descriptive, and the study lacks essential data including quantification. Further, the added value of some of the Cre lines is not completely clear.

Specific comments:
-None of the reported phenotypes are quantified in any way. The authors should include some quantifications to give an idea of the robustness of the phenotypes described.
We thank reviewer 2 for his comment. The reason why we didn't include quantifications is that the phenotype is 100% penetrant. We clearly noticed that every single *EphB1*$^{-/-}$ mouse, *EphB1*$^{\Delta Ex3/\Delta Ex3}$ mouse, *EphB1* cKO^{Vgat} mouse and *EphB1* cKO$^{dlx1/2}$ mouse clearly displayed the axon guidance deficits described in Figure 1. The phenotype is super robust and 100% consistent. We could tell by eye right away in a blind-to-genotype manner if a brain displayed the axon guidance deficits or not. However, we now added the number of animals for each experiment in the figure legend, and we added a table in Suppl. Fig. 12 describing the phenotype of each line. Since the slides are not usable anymore due to bleaching over the years, and since we don't have any of these crosses anymore, we were not able to add further details.

-The expression of EphB1 in the different cell compartments should be shown, either using the LacZ line or by in situ hybridization.
We reported relevant EphB1 expression in the mouse embryo brain in our previous article [6]. However, we agree with reviewer 2 that it was important to assess EphB1 expression at E14.5 in the different cell types of the mouse embryo, right before the axon guidance process we are interested in. For that, we collaborated with Stefano Berto, a bioinformatician, to analyze the published single cell RNAseq dataset of whole embryonic mouse brains [5]. We found that EphB1 is strongly expressed in the expected cell types, such as forebrain GABAergic and glutamatergic cells, or oligodendrocytes. Interestingly, using this dataset, we discovered that EphB1 is also expressed in angioblasts, endothelial cells precursors. However, our work showed that EphB1 deletion from endothelial cells did not phenocopy the defects observed in *EphB1*$^{-/-}$ mice. We now added this detailed analysis of EphB1 expression in Suppl. Fig.11.

© 2023. Published by The Company of Biologists under the terms of the Creative Commons Attribution License (https://creativecommons.org/licenses/by/4.0/).

-Similarly, the loss of EphB1 expression in these compartments following Cre excision should be shown on tissue sections.

Reviewer 1 had a similar request. We found misguided long-range striatal GABAergic projections, but EphB1 deletion from Drd1+ and Drd2+ cells did not phenocopy the *EphB1*$^{-/-}$ mice. However, Drd1-Cre and Drd2-Cre might not recombine early enough to affect the axon guidance process we're interested in, therefore preventing us from drawing a clear conclusion. Due to loss of funding for this study since a while and to Dr. Assali's departure to France for a new job in research in July 2022, it was not possible for us to confirm EphB1 recombination using the different cre lines. We were forced to stop the numerous and expensive crosses of this project and to cryopreserve the sperm from the EphB1 floxed mice. However, to address this issue the best way we can, we crossed the Drd1-Cre and Drd2-Cre mice with Ai14 reporter mice and found clear recombination at E14.5. This finding is now included in the Suppl. Fig. 10 in our revised manuscript. The Drd1-Cre and Drd2-Cre were the most important cre lines, since we found misguided long-range GABAergic projections from the striatum. The other Cre lines that we used have been extensively characterized in other publications [1-4]. In addition, we collaborated with Stefano Berto, a bioinformatician, to analyze the published single-cell RNAseq dataset of whole embryonic mouse brains [5] and confirmed expression of Tek (Tie-Cre), Cspg4 (Cspg4-Cre), Sst (Sst-Cre) at E14.5 in the appropriate cell types. The results of this analysis have been added to our revised manuscript in Suppl. Fig. 11.

-The added value of using Cspg4-Cre or Tie2-Cre is not completely clear, this part seems a bit distinct from the rest of the study.

EphB1 deletion using the Dlx1/2- and Vgat-Cre lines phenocopied the defects found in the *EphB1*$^{-/-}$ mice. This finding strongly suggested a role of EphB1 in GABAergic neurons. However, dlx and Vgat are not only expressed in GABAergic neurons early on, but they're also expressed in endothelial cells and dlx2 is expressed in a pool of progenitors giving rise to oligodendrocyte precursor cells (OPCs), known to migrate along blood vessels. EphB1 is expressed in OPCS, and using the single cell RNAseq dataset from E14.5 embryos, we found that EphB1 is also expressed in angioblasts. This suggests that using the dlx and Vgat-Cre lines, EphB1 is likely deleted from endothelial cells and OPCs. Therefore, EphB1 could have been playing a role in these latter cells, to repulse ephrinB-expressing axons. To rule out the possibility of a function of EphB1 in these endothelial or OPCs cells, we used the Tie2-Cre line to delete EphB1 from endothelial cells and NG2-Cre line to delete EphB1 from OPCs and mural cells. We didn't find any deficits. This analysis seemed important to us for understanding the mechanism underlying our phenotype, which is why we've included it in the manuscript.

Further, the fact that axon bundles follow vasculature in the EphB1 cKO could be due to a role of blood vessels as non-specific barriers that wandering axons « hit » and follow. In my opinion it does not necessarily imply that EphB1 plays an active role in promoting avoidance of blood vessels. More data is needed to fully support this notion.

We agree with reviewer 2 that we can't exclude that possible explanation of the phenotype. We've added a sentence to the revised discussion acknowledging the possibility that the blood vessel phenotype is not related to active EphB1-mediated avoidance of blood vessels. Future studies will be needed to better understand the axon/blood vessel phenotype in the EphB1 mutants, as the reviewer notes.

-In the discussion the authors focus on endothelial-specific ephrinB2, but they do not discuss the role of ephrinB2 (or other ephrins) as ligands expressed in glutamatergic cortical neurons. Do ephrin mutants (conditional and /or constitutive) exhibit axon guidance defects similar to EphB1? This point should be added in the discussion.

We agree with the reviewer that this is an important point to add to the discussion. We now added the following paragraph to our discussion in the revised manuscript:

"EphB1 mediates many of its biological functions through bidirectional signaling induced by contact-mediated binding to ephrins. EphrinB2 is strongly expressed in the developing cortex and striatum [2], and we previously showed that conditional deletion of ephrinB2 in Nestin-Cre mice, where recombination includes cortical glutamatergic neurons, did not phenocopy the axon guidance deficits found in EphB1$^{-/-}$ mice [2]. Conditional deletion of ephrinB2 using the FoxG1-Cre line produced thalamo-cortical axon guidance deficits similar to those observed in EphB1$^{-/-}$;EphB2$^{-/-}$ mice, but did not phenocopy the cortical axon guidance deficits found in EphB1$^{-/-}$ mice and in EphB1$^{-/-}$;EphB2$^{-/-}$ mice. EphrinB3, also expressed in the cortex and hippocampus, has been shown to control corticospinal tract axon guidance [44], but future studies will be needed to assess its

© 2023. Published by The Company of Biologists under the terms of the Creative Commons Attribution License (https://creativecommons.org/licenses/by/4.0/).

possible role in the EphB1 cKO^{Vgat} phenotype."

Reviewer 3 Advance Summary and Potential Significance to Field:
In general, the Eph family is the largest family of tyrosine kinase receptors in the brain and plays fundamental roles in virtually all neuro-developmental processes. Due to their complex biology (multiple overlapping expression patterns and the potential for bi-directional signaling makes this family a challenge to study. Therefore, the work in this manuscript is highly appreciated in which a conditional KO is generated and with a multitude of different Cre lines investigated.

Defects in a knockout of the guidance receptor EphB1 have been noted but the mechanisms behind remained unknown. The approaches taken by the authors are a good way forward to elucidate these.

Reviewer 3 Comments for the Author:
A full KO of the receptor EphB1 results in axon guidance defects of axons from cells in the deeper layers of the somatosensory cortex; however, the cell types affected and the precise role/mechanism of EphB1 in this processes deduced from the observed defects had not be described in further detail so far. Since members of the Eph family signal/function bi-directionally, multiple mechanisms to explain these defects appear possible.

The authors generated a novel floxed EphB1 allele and bred this with a massive battery of different Cre lines, and did a careful and detailed analysis to gain further insight into this interesting question.

Conditional KO of EphB1 in excitatory neurons of the deep layers of the somatosensory cortex did not lead to guidance defects, but a cKO of EphB1 in GABAergic neurons of the striatum shows guidance defects mimicking the full KO. Thus, in this particular cKO defects of axons of excitatory neurons from the somatosensory cortex now were observed. This suggests a non-cell autonomous defect from a cKO of EphB1 in striatal GABAergic but not cortical GABAergic neurons onto excitatory axons/neurons from the somatosensory cortex, i.e. some disturbed fasciculation is discussed.

Surprisingly, a cKO of EphB1 in dopaminergic neurons of the striatum did not lead to these guidance defects.

Investigation of a possible role of EphB1 in endothelial cells and other cell types of the vasculature - approached using further Cre lines did not elucidate the mechanisms of disturbed axon fascicle formation.

Comments

Overall, the topic, the manuscript, and the large number of cre lines used appear quite complex, making it wishful to include some schemes of the results.
This is a good suggestion. We now included a scheme that summarizes our finding in Supplemental Figure 13.

I find it surprising that very little attention is being paid to the role of ephrinBs in this manuscript. Occasionally but more spontaneously, an apparent (co-) expression on axon tracts and else structures with EphBs is noted, but overall a systemic inclusion of expression patterns and their possible role in explaining the EphB1 defects is lacking, but very wishful to see, that is **some key expression data for ephrinBs appear necessary to be included in this manuscript**.
We previously reported ephrinB2 spatial expression at E13 [6], using the ephrinB2-LacZ mice. EphrinB2 is strongly expressed in the developing cortex and developing striatum. We now added this information to our revised manuscript. There are 3 ephrinBs expressed in the brain, ephrinB 1-3. None of them have good antibodies. To precisely assess ephrinB (and ephrinA) expression at E14.5 in the different cell types of the mouse embryo, right before the axon guidance process we are interested in, we analyzed the published single cell RNAseq dataset of embryonic mouse brains [5], now included in the Suppl. Fig. 11. In our first submission, we discussed a potential role of ephrinBs in developing vasculature. In addition to that, we now added another paragraph in the

© 2023. Published by The Company of Biologists under the terms of the Creative Commons Attribution License (https://creativecommons.org/licenses/by/4.0/).

discussion about ephrinBs' potential role in our phenotype.

It is claimed that little is known about brain vasculature and CNS axon guidance, in fact there is quite an extensive literature which should be referred to properly.
We now removed this inappropriate statement, but we've kept the articles we cited in the discussion about this general topic.

1. Liang, H., S. Hippenmeyer, and H.T. Ghashghaei, *A Nestin-cre transgenic mouse is insufficient for recombination in early embryonic neural progenitors*. Biol Open, 2012. **1**(12): p. 1200-3.
2. Potter, G.B., et al., *Generation of Cre-transgenic mice using Dlx1/Dlx2 enhancers and their characterization in GABAergic interneurons*. Mol Cell Neurosci, 2009. **40**(2): p. 167-86.
3. Saunders, A., K.W. Huang, and B.L. Sabatini, *Globus Pallidus Externus Neurons Expressing parvalbumin Interconnect the Subthalamic Nucleus and Striatal Interneurons*. PLoS One, 2016. **11**(2): p. e0149798.
4. Kisanuki, Y.Y., et al., *Tie2-Cre transgenic mice: a new model for endothelial cell-lineage analysis in vivo*. Dev Biol, 2001. **230**(2): p. 230-42.
5. La Manno, G., et al., *Molecular architecture of the developing mouse brain*. Nature, 2021. **596**(7870): p. 92-96.
6. Robichaux, M.A., et al., *EphB receptor forward signaling regulates area-specific reciprocal thalamic and cortical axon pathfinding*. Proc Natl Acad Sci U S A, 2014. **111**(6): p. 2188-93.

Third decision letter

MS ID#: DEVELOP/2022/201439

MS TITLE: EphB1 controls proper long-range cortical axon guidance through a cell non-autonomous role in GABAergic cells

AUTHORS: Ahlem Assali, George Chenaux, Jennifer Y Cho, Stefano Berto, Nathan A Ehrlich, and Christopher Cowan

I have now received all the referees' reports on the above manuscript, and have reached a decision. The referees' comments are appended below, or you can access them online: please go to BenchPress and click on the 'Manuscripts with Decisions' queue in the Author Area.

As you will see, the referees express considerable interest in your work, but still have some significant criticisms and recommend a substantial revision of your manuscript before we can consider publication. If you are able to revise the manuscript along the lines suggested, which may involve further experiments, I will be happy receive a revised version of the manuscript.

Reviewer 1 remains concerned about the lack of validation of expression patterns. This reviewer notes you cannot rule out the possibility that the lack of a phenotype is due to a failure of gene deletion. While in situ or antibody staining is the gold standard for such a validation, you have used Ai14 for some Cre drivers (Drd1 and 2; Tie2), which can at least address concerns that the Cre mouse line was working in your hands. In addition, you include valuable RNA validation for other Cre drivers (Emx and Vgat). Thus, the main conclusion of the paper regarding the non-cell autonomous role in GABAergic neurons to influence axons is sound. However, there are several Cre lines (Nkx-Cre; SST-Cre; Cspg1-Cre) for which you conclude there is no phenotype, but which still lack validation or evidence that the Cre is working in your hands. Even though these are well characterized lines, I agree with Reviewer 1 that without validation of the Eph deletion or at a minimum use of a reporter, one cannot convincingly draw conclusions about negative data. Sometimes mice don't behave as expected even if they have been well characterized in the literature). Thus, the conclusion that Eph is dispensable in PV and SST or OPCs and mural cells is not sufficiently strong without these data. I note that reviewer 2 initially noted that having all these Cre lines was not necessary. As such, for us to proceed with considering the manuscript at

© 2023. Published by The Company of Biologists under the terms of the Creative Commons Attribution License (https://creativecommons.org/licenses/by/4.0/).

Development, I strongly encourage you to remove these data from the manuscript. This will require revising some smaller conclusions of the paper as well. Further, you should include in the discussion the limitation that in some cases Cre activity is monitored only by a reporter and not by direct investigation of the marker expression.

Beyond this, in your revised manuscript please address several additional points. Reviewer 3 indicates that you should revise the model and figure legend for supplemental Fig 13. I note the method section specifies that some Cre lines were crossed to Ai14 but this is not true for some lines. Finally, please consider to revise the order of figures and supplemental figures/panels to better match the order in which they are presented in the text. Some figures being out of order is ok but it seems a lot. For example, SuppFig1 should be moved to a later supplement for when you introduce generation of these mice. As another example, for Figures 4 and 5 this may also involve reorganizing figure panels (and removing panels as indicated above).

Your revised paper may be re-reviewed by one or more of the original referees, and acceptance of your manuscript will depend on your addressing satisfactorily the reviewers' major concerns. If it would be helpful, you are welcome to contact us to discuss your revision in greater detail. Please include in your submission, details of how you have modified the manuscript to address remaining concerns.

Please attend to all of the reviewers' comments and ensure that you clearly highlight all changes made in the revised manuscript. Please avoid using 'Tracked changes' in Word files as these are lost in PDF conversion. I should be grateful if you would also provide a point-by-point response detailing how you have dealt with the points raised by the reviewers in the 'Response to Reviewers' box. If you do not agree with any of their criticisms or suggestions please explain clearly why this is so.

Reviewer 1

Advance summary and potential significance to field

This paper potentially provides a novel non-cell autonomous mechanism by which EphB1 plays in cortical axon development.

Comments for the author

In this revised manuscript, Assali et al. did not address the major concern regarding the first submission. Validation of the gene deletion is essential especially when there is no obvious phenotypes. Since the successful gene deletion is influenced both by the efficiency of Cre expression and the efficiency of the floxed gene locus, just showing the reporter expression is not sufficient.

Reviewer 2

Advance summary and potential significance to field

The authors have addressed my comments.

Comments for the author

The authors have addressed my comments.

Reviewer 3

Advance summary and potential significance to field

as made in first review

© 2023. Published by The Company of Biologists under the terms of the Creative Commons Attribution License (https://creativecommons.org/licenses/by/4.0/).

Comments for the author

the authors address my concerns and provide some additional information the scheme summarizing their findings (Suppl Fig 13) I actually found underwhelming and I think needs a re-drawing and better legend

---

Third revision

Author response to reviewers' comments

This paper potentially provides a novel non-cell autonomous mechanism by which EphB1 plays in cortical axon development.

Reviewer 1 Comments for the author
In this revised manuscript, Assali et al. did not address the major concern regarding the first submission. Validation of the gene deletion is essential especially when there is no obvious phenotypes. Since the successful gene deletion is influenced both by the efficiency of Cre expression and the efficiency of the floxed gene locus, just showing the reporter expression is not sufficient.
- We now removed from the manuscript the data from the lines we did not validate: the dlx1/2, Sst, Nkx2.1, Cspg4 data.

Reviewer 2 Advance summary and potential significance to field
The authors have addressed my comments.

Reviewer 2 Comments for the author
The authors have addressed my comments.
- Thank you.

Reviewer 3 Advance summary and potential significance to field as made in first review

Reviewer 3 Comments for the author
the authors address my concerns and provide some additional information
- Thank you.

the scheme summarizing their findings (Suppl Fig 13) I actually found underwhelming and I think needs a re-drawing and better legend

- We have revised this supplemental figure and legend to improve and clarify the summary of our paper findings.

Fourth decision letter

MS ID#: DEVELOP/2022/201439

MS TITLE: EphB1 controls proper long-range cortical axon guidance through a cell non-autonomous role in GABAergic cells

AUTHORS: Ahlem Assali, George Chenaux, Jennifer Y Cho, Stefano Berto, Nathan A Ehrlich, and Christopher Cowan
ARTICLE TYPE: Research Article

I am happy to tell you that your manuscript has been accepted for publication in Development, pending our standard ethics checks.

© 2023. Published by The Company of Biologists under the terms of the Creative Commons Attribution License (https://creativecommons.org/licenses/by/4.0/).
